# Myoglobinopathy is an adult-onset autosomal dominant myopathy with characteristic sarcoplasmic inclusions

**DOI:** 10.1038/s41467-019-09111-2

**Published:** 2019-03-27

**Authors:** Montse Olivé, Martin Engvall, Gianina Ravenscroft, Macarena Cabrera-Serrano, Hong Jiao, Carlo Augusto Bortolotti, Marcello Pignataro, Matteo Lambrughi, Haibo Jiang, Alistair R. R. Forrest, Núria Benseny-Cases, Stefan Hofbauer, Christian Obinger, Gianantonio Battistuzzi, Marzia Bellei, Marco Borsari, Giulia Di Rocco, Helena M. Viola, Livia C. Hool, Josep Cladera, Kristina Lagerstedt-Robinson, Fengqing Xiang, Anna Wredenberg, Francesc Miralles, Juan José Baiges, Edoardo Malfatti, Norma B. Romero, Nathalie Streichenberger, Christophe Vial, Kristl G. Claeys, Chiara S. M. Straathof, An Goris, Christoph Freyer, Martin Lammens, Guillaume Bassez, Juha Kere, Paula Clemente, Thomas Sejersen, Bjarne Udd, Noemí Vidal, Isidre Ferrer, Lars Edström, Anna Wedell, Nigel G. Laing

**Affiliations:** 1 Neuropathology Unit, Department of Pathology and Neuromuscular Unit, Department of Neurology, IDIBELL-Hospital de Bellvitge, Hospitalet de Llobregat, Barcelona, 08907 Spain; 2 Department of Molecular Medicine and Surgery, Science for Life Laboratory, Karolinska Institutet, Stockholm, SE-17176 Sweden; 3 Centre for Inherited Metabolic Diseases, Karolinska University Hospital, Stockholm, SE-17177 Sweden; 4 Centre for Medical Research, University of Western Australia, Harry Perkins Institute of Medical Research, Perth, 6000 WA Australia; 5 Neurology Department and Instituto de Biomedicina de Sevilla, Hospital Universitario Virgen del Rocío, Seville, 41013 Spain; 6 Department of Biosciences and Nutrition, Science for Life Laboratory, Karolinska Institutet, Stockholm, SE-14157 Sweden; 7 Clinical Research Centre, Karolinska University Hospital, Huddinge, SE-17177 Sweden; 8 Department of Life Sciences, University of Modena and Reggio Emilia, Modena, 41121 Italy; 9 Department of Chemical and Geological Sciences, University of Modena and Reggio Emilia, Modena, 41121 Italy; 10 School of Molecular Sciences, The University of Western Australia, 35 Stirling Highway, Crawley, 6009 WA Australia; 11 ALBA Synchrotron Light Source, Cerdanyola del Vallès, Barcelona, 08290 Spain; 12 Division of Biochemistry, Department of Chemistry, Vienna Institute of BioTechnology, BOKU—University of Natural Resources and Life Sciences, Vienna, A-1180 Austria; 13 School of Human Sciences, The University of Western Australia, Perth, 6000 Western Australia Australia; 14 Victor Chang Cardiac Research Institute, Darlinghurst, 2010 NSW Australia; 15 Unitat de Biofísica, Departament de Bioquímica i de Biologia Molecular, Facultat de Medicina, Universitat Autònoma de Barcelona, Bellaterra, 08193 Spain; 16 Department of Molecular Medicine and Surgery, Karolinska Institutet, and Department of Clinical Genetics, Karolinska University Hospital, Solna, Stockholm, SE-17176 Sweden; 17 Department of Women’s and Children’s Health, Karolinska Institutet, Stockholm, SE-17177 Sweden; 18 Department of Medical Biochemistry and Biophysics, Karolinska Institutet, Stockholm, SE-17177 Sweden; 19 Neurology Department, Hospital Son Espases, Palma de Mallorca, 07120 Spain; 20 Neurology Department, Hospital Verge de la Cinta, Tortosa, 43500 Spain; 21 Université Sorbonne, UPMC Univ Paris 06, INSERM; UMRS974, CNRS FRE3617, Center for Research in Myology, GH Pitié-Salpêtrière, 47 Boulevard de l’hôpital, 75013 Paris, France; 22 Centre de Référence de Pathologie Neuromusculaire Paris-Est, Institut de Myologie, GHU Pitié-Salpêtrière, Assistance Publique-Hôpitaux de Paris, Paris, 75013 France; 23 Centre de Pathologie et Neuropathologie Est, Hospices Civils de Lyon; Université Claude Bernard Lyon1, Institut NeuroMyogène CNRS UMR 5310—INSERM U1217; Institut NeuroMyogène, Villeurbanne, 69677 France; 24 Electromyographie—Groupement Hospitalier Est, Hospices Civils de Lyon, 69677 France; 25 Department of Neurology, University Hospitals Leuven, Leuven, 2333 Belgium; 26 KU Leuven—University of Leuven, Laboratory for Muscle diseases and Neuropathies, Department of Neurosciences, Experimental Neurology, Leuven, 2333 Belgium; 27 Department of Neurology, Leiden University Medical Center, Leiden, 2333 The Netherlands; 28 KU Leuven—University of Leuven, Laboratory for Neuroimmunology, Department of Neurosciences, Experimental Neurology, Leuven, 2333 Belgium; 29 Department of Pathology, Antwerp University Hospital, Edegem, 2650 Belgium; 30 Laboratory of Neuromuscular Pathology, Institute Born-Bunge, University of Antwerp, Wilrijk, 2610 Belgium; 31 Department of Pathology, Radboud University Medical Center, Nijmegen, 6525 The Netherlands; 32 Neuromuscular Reference Center, Henri Mondor University Hospital AP-HP, INSERM U955, Team 10, Biology of the Neuromuscular System, East-Paris University (UPEC), Paris, 94010 France; 33 Molecular Neurology Research Program, University of Helsinki and Folkhälsan Institute of Genetics, Helsinki, 00014 Finland; 34 School of Basic and Medical Biosciences, King’s College London, London, WC2R2LS UK; 35 Neuromuscular Research Center, Tampere University Hospital, University of Tampere, Tampere, 33521 Finland; 36 Folkhälsan Genetic Institute, University of Helsinki, Helsinki, 00250 Finland; 37 Neurology Department, Vasa Central Hospital, Vasa, Finland Neuromuscular Research Center, Tampere University Hospital, University of Tampere, Tampere, 65100 Finland; 38 Department of Pathology and Experimental Therapeutics, University of Barcelona, CIBERNEDHospitalet de LLobregat, Barcelona, 08907 Spain; 39 Center for Molecular Medicine, Karolinska Institutet, Stockolm, 17177 Sweden

## Abstract

Myoglobin, encoded by *MB*, is a small cytoplasmic globular hemoprotein highly expressed in cardiac myocytes and oxidative skeletal myofibers. Myoglobin binds O_2,_ facilitates its intracellular transport and serves as a controller of nitric oxide and reactive oxygen species. Here, we identify a recurrent c.292C>T (p.His98Tyr) substitution in *MB* in fourteen members of six European families suffering from an autosomal dominant progressive myopathy with highly characteristic sarcoplasmic inclusions in skeletal and cardiac muscle. Myoglobinopathy manifests in adulthood with proximal and axial weakness that progresses to involve distal muscles and causes respiratory and cardiac failure. Biochemical characterization reveals that the mutant myoglobin has altered O_2_ binding, exhibits a faster heme dissociation rate and has a lower reduction potential compared to wild-type myoglobin. Preliminary studies show that mutant myoglobin may result in elevated superoxide levels at the cellular level. These data define a recognizable muscle disease associated with *MB* mutation.

## Abstract (web summary)

Myoglobin is a hemeprotein that reversibly binds oxygen and gives muscle its red color. Here, the authors report a genetic variant in the MB gene that associates with myoglobinopathy, an autosomal dominant progressive myopathy, and altered oxygen binding properties of the mutant protein.

## Introduction

Myoglobin, the pigment that gives muscle its red color, is a small cytoplasmic globular hemoprotein highly expressed in cardiac and oxidative skeletal muscle fibers^1^.

By reversibly binding O_2_, myoglobin buffers intracellular O_2_ concentrations, facilitates intracellular O_2_ transport and serves as a reservoir of oxygen during hypoxic and anoxic conditions^1–3^. In addition, myoglobin is implicated in vivo in the control of redox pathways in skeletal and cardiac myocytes, acting as scavenger of reactive oxygen species (ROS) and nitric oxide^4,5^.

Myoglobin was the first protein for which a three-dimensional structure was determined by X-ray crystallography^6^. The backbone of myoglobin consists of eight α-helices, assigned the letters A to H, that wrap around a central pocket containing a heme group, a porphyrin ring that contains a central bound iron atom that is normally in the ferrous oxidation state. Its heme active site is responsible for reversible binding to various ligands including oxygen, carbon monoxide and nitric oxide^7^.

Myoglobin is encoded by the myoglobin gene (*MB*) on human chromosome 22q12.3^7^.

Here we describe myoglobinopathy, a disease caused by a recurrent *MB* mutation found in 14 patients from six unrelated European families suffering from an autosomal dominant myopathy with variable cardiac involvement and characteristic sarcoplasmic inclusions in skeletal and cardiac muscle. We show that mutation of *MB* alters the kinetics and thermodynamics of O_2_ binding and may result in elevated superoxide levels.

## Results

### The clinical phenotype of myoglobinopathy

**Fig. 1:**
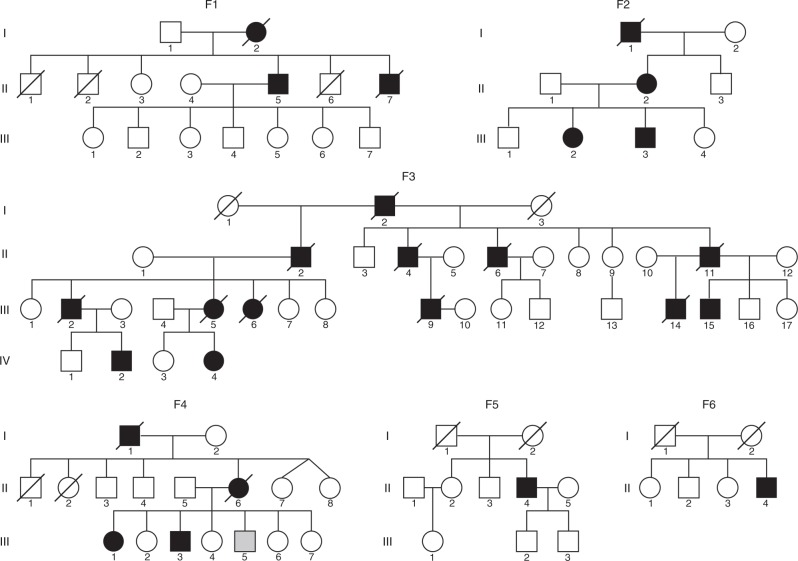
Pedigrees from the six families affected with myoglobinopathy. F1 and F2 Spanish, F3: Swedish, F4 and F5: French, F6: Dutch. Squares represent males; circles, females; filled black symbols indicate affected individuals; filled gray symbol, clinically affected, but not molecularly tested individuals; and slash, deceased

We studied 14 patients from six unrelated European families (F1–F6), (Fig. 1), suffering from an autosomal dominant myopathy with variable cardiac involvement, identified by their highly characteristic features on muscle biopsies. The clinical presentation was homogeneous among all patients and is summarized in Table 1. Age of onset ranged between 33 and 49 years. Initial symptoms were proximal lower limb, pelvic girdle, and axial muscle weakness, manifesting with difficulties climbing stairs, rising from squatting and standing up from the lying position. Two patients had, in addition, weakness and atrophy of thenar muscles from disease onset. Over the following years, the disease slowly progressed to involve distal leg and hand muscles, proximal muscles of upper limbs, and neck muscles. Five patients complained of dysphagia. Facial and extraocular muscles were not involved. Progression was slow; most patients developed respiratory failure requiring nocturnal non-invasive ventilatory support 10 years after disease onset and they became wheelchair dependent 15–20 years after disease onset. Cardiac involvement, revealed by ultrasound, magnetic resonance imaging (MRI) or post mortem myocardium examination, was observed in six individuals. Cardiac MRI in patient F2, II:2 showed a dilated cardiomyopathy with extensive and diffuse areas of late gadolinium enhancement, indicative of fibrosis at the epicardium and mesocardium, but no involvement of the endocardium, (Supplementary Fig. 1). Six patients died between 18 and 30 years after disease onset, from respiratory and/or cardiac failure.

**Table 1:** Phenotypic features of myoglobinopathy

	Family 1	Family 2	Family 3	Family 4	Family 5	Family 6
Inheritance pattern	AD	AD	AD	AD	unknown	unknown
Myoglobin mutation	His98Tyr	His98Tyr	His98Tyr	His98Tyr	His98Tyr	His98Tyr
Country of origin	Spain	Spain	Sweden	France	France	Netherlands
No. of patients	2	1	6	3	1	1
Mean age of onset (range)	37.5 (36–39)	38	44.5 (39–49)	46 (44–48)	40	33
Gender (female/male)	0/2	1/0	2/4	2/1	0/1	0/1
Initial symptoms	Proximal LL and axial weakness (2/2)	Proximal LL and axial weakness (1/1)	Distal hand weakness (2/6) Proximal LL and axial weakness (6/6)	Proximal LL and axial weakness (3/3)	Proximal LL weakness (1/1)	Proximal LL and axial weakness (1/1)
Symptoms at advanced disease						
Distribution of weakness	Proximal and axial > distal 4 EE	Proximal and axial > distal 4 EE	Proximal and axial > distal 4 EE	Proximal and distal 4 EE > axial	Proximal and axial > distal 4 EE	Proximal and axial > distal legs
Involvement of hand muscles	2/2	1/1	6/6	2/2**	1/1	0/1
Facial weakness	0/2	0/1	0/6	0/2	0/1	0/1
Muscle atrophy	2/2	1/1	6/6	2/2	0/1	1/1
Dysphagia	2/2	0/1	2/4	0/2	1/1	0/1
Respiratory insufficiency	2/2	1/1	2/6	1/2	1/1	1/1
Cardiac involvement*	2/2	1/1	2/6	0/2	1/1	0/1
Clinical outcome						
Mean age at wheelchair dependency	54	65	56 (4/6 patients wheelchair dependent, 2/6 ambulant)	66 (1/3 patients wheelchair dependent, 2/3 ambulant)	56	47
Mean age at death (range)	60.5 (54–67)	–	64 (58–71)	72	–	–
Laboratory studies						
CK fold elevation above normal levels	1.5–2	2	1.5–4	2.3–3.7	4	3.5–7
EMG	Myogenic with spontaneous activity at rest	Myogenic with spontaneous activity at rest	Myogenic with spontaneous activity at rest	Myogenic with spontaneous activity at rest	Myogenic with spontaneous activity at rest	Myogenic with spontaneous activity at rest

*As revealed by cardiac ultrasound, cardiac MRI, or by post mortem examination of cardiac muscle. ** Individual III:2 from Fam 4 is in the early stages of the disease and therefore not included in symptoms at advanced disease items. EE: extremities; LL: lower limbs

**Fig. 2:**
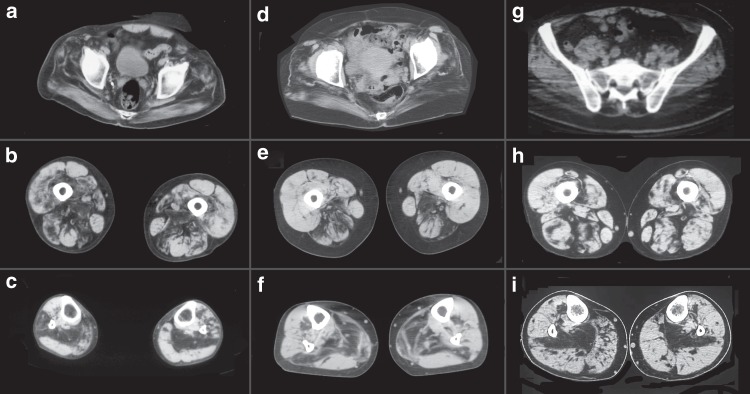
Muscle imaging in myoglobinopathy. Muscle CT scan from individuals F1, II: 7 (**a**–**c**); F4, II: 4 (**d**–**f**); and F5, II: 6 (**g**–**i**) at the pelvic (**a**, **d** and **g**), mid-thigh (**b**, **e**, and **h**), and mid-leg (**c**, **f**, and **i**). At the pelvis, there is involvement of the gluteus maximus, medius, and minimus. At mid-thigh, there is preferential involvement of the posterior compartment, specially, of the adductor magnus, biceps femoris, and semimembranosus. At the mid-leg, the soleus is the first and most-affected muscle

Serum CK levels were normal to mildly increased, except for a single individual who showed CK levels up to sevenfold above the upper normal limit. EMG was consistent with a myopathy with spontaneous activity at rest. Nerve conduction studies were normal. Muscle imaging studies showed a consistent pattern characterized by fatty degenerative changes in paraspinal and gluteal muscles, especially gluteus maximus and medius. At mid-thigh, there was preferential involvement of the adductor magnus, semimembranosus, long head of biceps femoris and vastus intermedius, except in individual F6, II:4 who showed predominant involvement of the anterior compartment. At the mid-leg, the soleus was always the first and most affected muscle (Fig. 2).

### Muscle pathology features associated with MB mutation

**Fig. 3:**
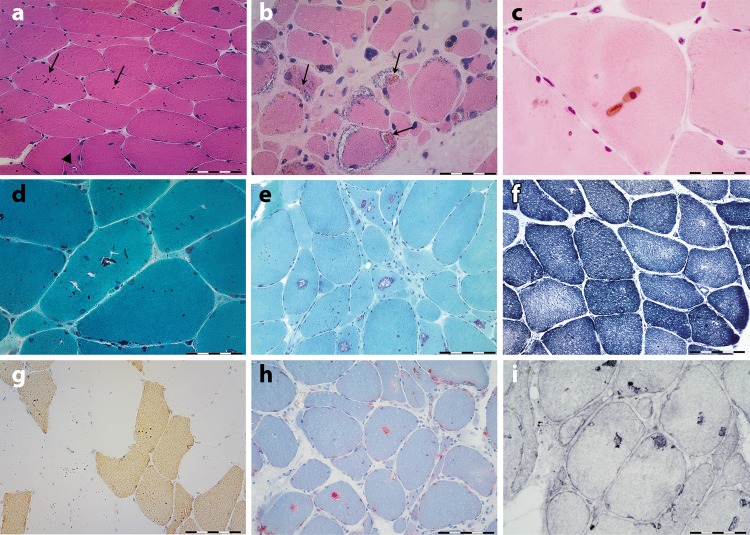
Histochemical features of myoglobinopathy. **a** Anterior tibialis muscle biopsy from individual F3, III: 15, 10 years prior to the onset of symptoms, stained with hematoxylin and eosin, showing several rounded brown inclusions (arrows) (sarcoplasmic bodies) in the majority of myofibers and very small vacuoles in some myofibers (arrowhead in **a**). **b**, **c** Biceps brachii from individual F1, II: 7, 15 years after disease onset. **b** Note the presence of collections of sarcoplasmic bodies within the rimmed vacuoles. **d**, **e** Sarcoplasmic bodies appear red on modified Gomori trichrome stain. **e** In muscle biopsies with more advanced pathological lesions, large numbers of rimmed vacuoles are observed. **f** No major architectural changes are seen on NADH reaction, apart from lack of oxidative activity at the site of vacuoles. **g** Fast myosin immunohistochemistry demonstrate the presence of sarcoplasmic bodies in both type 1 (slow) and 2 (fast) myofibers. **h** Myofiber regions containing vacuoles display strong phosphatase activity, and LAMP1 **i** immunoreactivity. Scale bar in **a**, **e**, **f**, **g**, and **h** = 50 µm; scale bar in **b**, **c**, **d**, and **i** = 20 µm

Muscle biopsies showed characteristic sarcoplasmic bodies in both Type 1 and Type 2 myofibers in all studied patients, including pre-symptomatic individuals, irrespective of the site of the biopsy (Fig. 3 and Supplementary Table 1). The sarcoplasmic bodies were rounded or oval, appeared brown on hematoxylin and eosin and red on the modified Gomori’s trichrome stain. They had a glassy appearance and were easily visualized with all stains and even in non-stained sections.

**Fig. 4:**
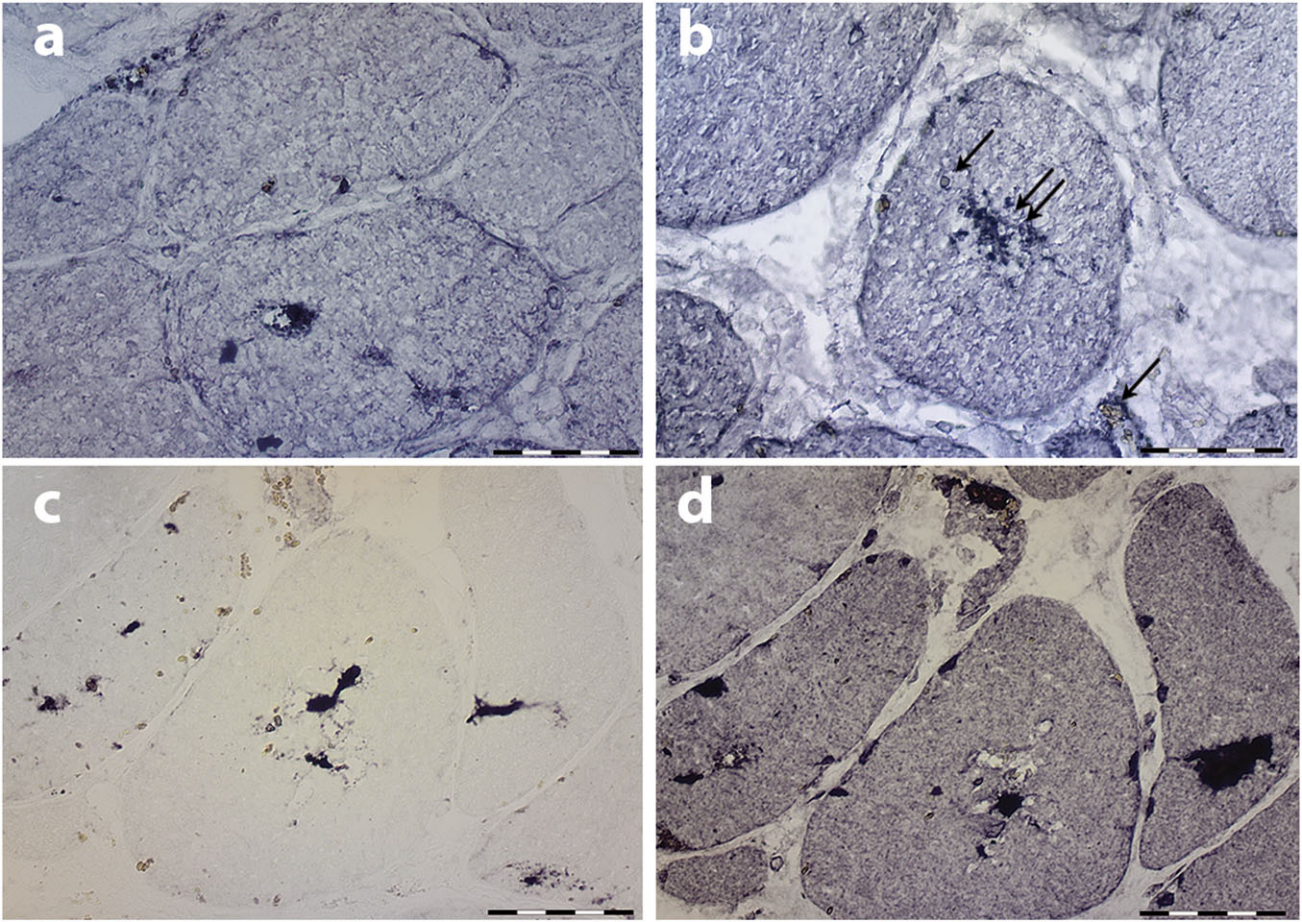
Immunohistochemical features of myoglobinopathy. **a**, **b** Small myoglobin aggregates are observed in some myofiber regions and in some sarcoplasmic bodies (double arrow in **b**), but not in others (arrow in **b**). **c** p62 and ubiquitin **d** immunoreactivity is observed in some myofiber regions, and in some, but not in all sarcoplasmic bodies indicating protein aggregates. Scale bars = 20 µm

**Fig. 5:**
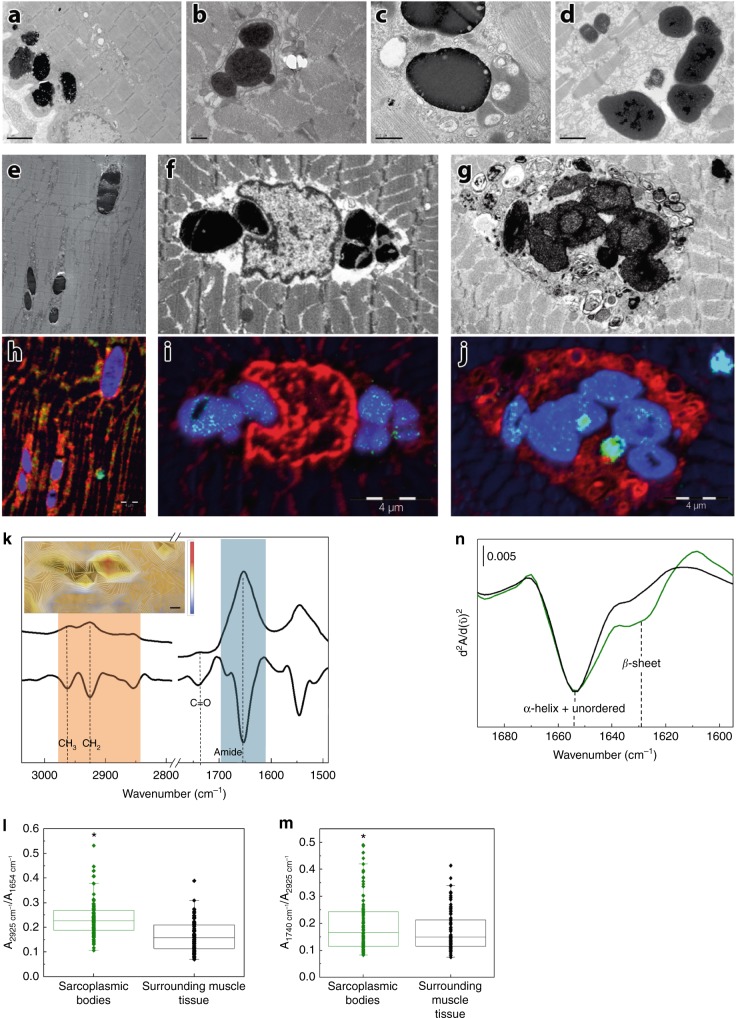
Characterization of sarcoplasmic bodies, the morphological hallmark of myoglobinopathy. Electron micrographs **a**–**d** showing collections of highly electron-dense bodies with some less dense material at their periphery. The sarcoplasmic bodies are seen under the sarcolemma (**a**) and often next to the nuclei. Some sarcoplasmic bodies are surrounded by a membrane (**b**). Sarcoplasmic bodies of different electron densities near several vesicular structures (**c**). Sarcoplasmic bodies observed in the cardiac muscle obtained post mortem from individuals F1, II:7 (**d**) and F3, III:5. Electron micrographs (**e**–**g**) and the corresponding NanoSIMS images (**h**–**j**). Blue indicates sulfur (^32^S), red phosphorus (^31^P), and green (^56^Fe), respectively. Sarcoplasmic bodies interspersed between the myofibrils (**e**), next to nuclei (**f**), or inside an autophagic vacuole (**g**). Note the high-sulfur signal in the sarcoplasmic bodies (**h**–**j**), and the iron signal (green dots within the sarcoplasmic bodies in **i**, **j**). Scale bar in **a** = 2 µm, **b**  = 5 µm, **c** = 0.5 µm, **d** = 1 µm, **h**–**j** = 4 µm. **k** Typical µFTIR spectra and their second derivative of the muscle tissue where the lipid region has been highlighted in orange and the protein region in blue; the inset shows the lipid/protein ratio (calculated from the Infrared spectra) on an optical image of a tissue section with sarcoplasmic bodies. The color bar represents intensity of the ratio: blue and red mean low and high lipid content, respectively. The scale bar is four microns. **l** Box plot graphic of lipid/protein ratio (2925 cm^−1^/1654 cm^−1^). **m** Box plot graphics representing COOH/CH_2_ ratio indicating lipid oxidation (1739 cm^−1^/2925 cm^−1^). Ratios were calculated from the second derivative of the spectra of three different samples from three different patients (at least 10 sarcoplasmic bodies per patient). Boxplots denote the median (center line), interquartile range (box), whiskers that represents the most extreme data that are not >1.5x IQR from the edge of the box and outliers that are the points outside this range. T-tests were used to compare the sarcoplasmic body ratios with the surrounding tissue ratios and determine the *p* value (*p<0.005). **n** Infrared second derivative spectrum of the amide region of one sarcoplasmic body (green) showing an increase of β-sheet structures, indicating protein aggregation. Second derivative of the amide region corresponding to the tissue surrounding the sarcoplasmic bodies (black)

They exhibited autofluorescence emission in a wide range of visible laser excitation lines (Supplementary Fig. 2). Besides the sarcoplasmic bodies, the majority of muscle biopsies showed non-specific myopathic changes with increased myofiber size variation, increased numbers of internal nuclei and type 1 fiber predominance (Supplementary Table 1). Most muscle biopsies, particularly those showing advanced pathological lesions, showed cytoplasmic vacuoles filled with granular basophilic material, displaying strong acid phosphatase activity and lysosome-associated membrane protein 1 (LAMP1) immunoreactivity (Fig. 3), indicating that they contained lysosomes. No major architectural changes were observed on NADH, SDH, and COX reactions apart from the lack of oxidative activity at the site of vacuoles. Myoglobin, p62, and ubiquitin immunoreactivity was observed in abnormal myofiber regions containing vacuoles and in some but not all sarcoplasmic bodies (Fig. 4), demonstrating abnormal protein aggregation (Fig. 4). However, myoglobin immunostaining was not particularly useful for the diagnosis. Under electron microscopy, the inclusions appeared as very dense bodies measuring 0.3–2.5 µm, often located next to myonuclei or dispersed between myofibrils (Fig. 5a–d and Fig. 5e–g). Some were membrane bound, whereas others were not. Some were denser than others, likely reflecting different stages of the pathological process. Autophagic vacuoles with cellular debris, myelin figures, and filaments measuring 8–12 nm were additional findings. Sarcoplasmic bodies were present in respiratory and cardiac muscles obtained post mortem from individual F1, II:7 and in cardiac muscle from individual F3, III:5 (Fig. 5d and Supplementary Fig. 3), thus indicating that cardiomyopathy was a primary consequence of the disease and not secondary to respiratory failure.

### Identification of p.His98Tyr *MB* substitution in six families

To identify the molecular cause of this myopathy two independent groups used two different strategies. The Australian group used whole-exome sequencing of three affected individuals from two Spanish families (F1, II:5 and II:7 and F2, II:2), and gene prioritization of shared variants using skeletal muscle expression enrichment data from FANTOM5^8^. This identified the same heterozygous missense variant (c.292C>T, p.His98Tyr) in the myoglobin gene (*MB*) in all three affected individuals. The Swedish group, studying family F3, identified one region on chromosome 22 through genome-wide linkage analysis, with a statistically significant LOD score. Refinement of the peak with further markers and clarification of the disease state in family members, resulted in a maximum multipoint LOD score of 5.8 at D22S685 (Supplementary Fig. 4). Targeted capture and sequencing of genes within the linkage region in eight individuals, six affected and two unaffected, revealed in all the affected family members, the presence of the same *MB* (c.292C>T, p.His98Tyr) variant. Sanger sequencing confirmed segregation of the variant with the disease in all available family members and in three additional families (F4, F5, and F6). The variant involves a highly conserved residue in the proximity of the oxygen-binding heme group (Supplementary Fig. 5), it is absent in all unaffected relatives tested, is not present in the 1000genomes, ExAC (http://exac.broadinstitute.org/) or gnomAD ((http://gnomad.broadinstitute.org) data sets and was present on different haplotypes. Moreover, all in silico predictors suggested the observed substitution in *MB* is deleterious: MutationAssessor (medium functional impact, FI score 3.175), MutationTaster (disease-causing, *p*=0.999), PolyPhen-2 (probably damaging, score 1.000), Provean (deleterious, score −4.093) and SIFT (damaging, score 0.001). Thus, we have compelling evidence across six families that the same *MB* (c.292C>T, p.His98Tyr) variant is responsible for the observed myopathy.

Haplotype analysis using microsatellites within 3 Mbp of the *MB* gene indicated at least three different haplotypes within the six families, suggesting that the c.292C>T variant is a recurrent variant originating on different ancestral backgrounds (Supplementary Table 2). Furthermore, the analysis of haplotypes in family F5 showed that the affected and two unaffected siblings had identical alleles, suggesting that in the affected individual the variant probably arose de novo (Supplementary Table 2). No samples from the parents in family F5 were available for confirmation.

Myoglobin knockout mice have a binary phenotype. Two-thirds die in utero at ~ E9.5-E10.5^9^, but those that survive live to adulthood with little sign of functional effects, because of multiple compensatory mechanisms^10^. We therefore hypothesized a dominant gain of function as the cause of the disease in the families and used multiple methods to investigate the pathomechanism.

### NanoSIMS analysis of the sarcoplasmic bodies

Correlative electron microscopy and NanoSIMS (Nanoscale Secondary Ion Mass Spectrometry) analysis revealed high sulfur content and small iron Fe signals in the sarcoplasmic bodies (Fig. 5h–j). Of note, sulfur is a component of antioxidant systems of cells and reactive sulfur species are formed under conditions of oxidative stress^11,12^. Iron signals could be related to degradation of myoglobin and other metalloproteins inside the lysosomes^13^. Comparative NanoSIMS analysis of myoglobinopathy samples with one sample from a patient with Pompe disease containing electron-dense inclusions, two samples showing abundant lipofuscin and two muscle samples from patients affected with distal myopathies with rimmed vacuoles, revealed that the amount of Fe is higher in the myoglobinopathy sarcoplasmic bodies than in other similar inclusions.

### µFTIR analysis of muscle biopsy samples

Fourier transform infrared microscopy (µFTIR) analysis of muscle samples demonstrated increased carbonyl (ester) groups in the sarcoplasmic bodies indicating lipid oxidation (Fig. 5k–m). Moreover, some sarcoplasmic bodies showed a band at 1627 cm^−1^, which is characteristic of intermolecular β-sheet structures^14^ (Fig. 5n) and a feature typical of amyloid formation^15,16^. However, in vitro, only small amounts of non-fibrillar amyloid β-structure were detected (see Supplementary Note 1 and Supplementary Fig. 6). Non-fibrillar β-sheet aggregates have been previously described for other peptides and proteins including β-amyloid peptides related to Alzheimer’s disease^16^. Both NanoSIMS and µFTIR, therefore, suggest lipid oxidation in patients’ muscles contribute to sarcoplasmic body formation.

### Biochemical characterization

At the molecular level, the His98Tyr variant might impact on different MB functional properties, crucial to the physiological role: these include the interaction of MB with dioxygen, the affinity of MB protein scaffold to hemin, as well as the ability to keep the heme in the reduced state (Fe^2+^). In vivo, ferrous MB (Fe^2+^), which is the only myoglobin form that can bind and store O_2_, is prone to autoxidation^17^, leading to functionally inactive MetMB (Fe^3+^), which is eventually reduced back to ferrous MB by cytochrome *b*_*5*_^18,19^.

First, we evaluated whether the variant affects the affinity of the prosthetic group for the MB polypeptide matrix, as it has been demonstrated that mutations in the vicinity of the porphyrin ring can increase heme dissociation rates (*k*_-H_) by two-orders of magnitude^20–22^. At pH 7.0, *k*_-H_ of H98Y was five times higher compared with WT (Table 2 and Supplementary Fig. 7). Mutations of the corresponding residue (His97) in sperm whale myoglobin into small and hydrophobic (e.g., Ala, Val) or acidic (e.g., Glu, Asp) amino acids enhanced *k*_-H_^21,22^ owing to loss of electrostatic interaction between the side chain of His97 and the negatively charged heme propionate-7^21^. His98 of human MB contributes to modulating the solvent accessibility to the heme pocket. Upon exchange of His98 with Tyr, the interaction with the propionate is weakened; Tyr98 can still form H-bonds with propionate-7, nevertheless, *k*_-H_ is higher for p.His98Tyr compared with WT MB. Notably, this change in *k*_-H_ is in line with the His97Phe mutant of sperm whale myoglobin^21,22^, reflecting the similarity between Tyr and Phe in terms of steric hindrance. Our interpretation is further supported by the fact that at pH 5.0 the *k*_-H_ values for WT and p.His98Tyr are increased (1.44 ± 0.09 h^−1^ and 1.86 ± 0.05 h^−1^, respectively) and the difference between the two species becomes smaller, because of protonation of the heme propionates, resulting in diminished interaction of the heme group with the protein.

**Table 2:** Effect of mutation on biochemical properties of MB

	*K*_D, O2_ (µM)	*k*_-H_ at pH 7.0 (h^−1^) ^a^	*k*_-H_ at pH 5.0 (h^−1^) ^b^	*E*°’ by spectroelectr. (V vs SHE) ^c^	*E*°’ by SWV (V vs SHE) ^c^	*k*_OX_ at pH 7.0 (min^−1^)	Heme SASA (nm^2^) ^d^
WT	1.2 ± 1.1	0.22 ± 0.06	1.44 ± 0.09	+0.040 ± 0.005	−0.056 ± 0.015	1.96 ± 0.39	1.45 ± 0.04
His98Tyr	8.4 ± 2.5	1.19 ± 0.05	1.86 ± 0.05	+0.021 ± 0.005	−0.105 ± 0.015	1.97 ± 0.43	1.80 ± 0.05

Conditions: ^a^0.2 m phosphate buffer, pH 7.0, and 0.45 sucrose (37 °C); ^b^0.2 m acetate, pH 5.0, and 0.45 m sucrose (37 °C); ^c^0.2 m phosphate buffer, pH 7.0 (25 °C). ^d^Values obtained from MD simulations

**Fig. 6:**
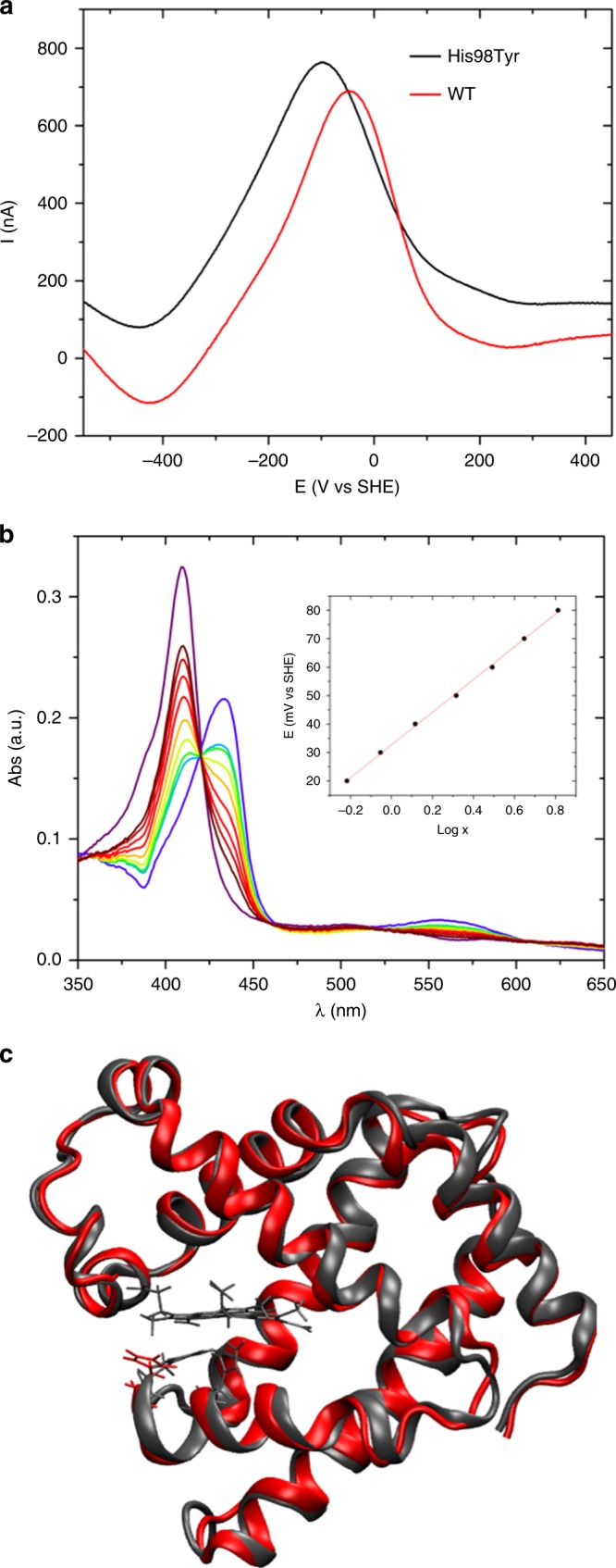
In vitro and in silico studies of WT and mutant MB. **a** Typical square wave voltammograms recorded for WT (black) and p.His98Tyr mutant (red) human myoglobin immobilized on a Au electrode in 20 mm phosphate buffer, pH 7.0, 25 °C. **b** Electronic spectra of p.His98Tyr mutant of human myoglobin obtained at various applied potentials E in spectroelectrochemical experiments carried out with an optical thin-layer electrochemistry cell at pH 7.0, 25 °C. The corresponding Nernst plot is shown in the inset, where *x* = [(A^max^_λred_−A_λred_)−(A^max^_λox_−A_λox_). **c** Overlay of cartoon representations of representative structures sampled within the MD simulations for native (red) and p.His98Tyr mutant (black) human myoglobin. His98 is also shown as stick with the same color coding. The heme group and Fe-ligand residue His94 is shown only for WT for sake of clarity

Next, we investigated the impact of the mutation on the oxidation state of the heme iron, both from a thermodynamic and kinetic point of view. Oxidized and reduced MB coexist in solution, and the ratio of the two forms is regulated thermodynamically by the reduction potential *E*°’ and kinetically by the protein autoxidation rate^17^, which in turn are influenced by pH, oxygen concentration, and temperature^23^. The autoxidation process is kinetically controlled under physiological conditions. We observed nearly identical autoxidation rate constants *k*_ox_ for WT and p.His98Tyr (see Table 2). Moreover, the spectral features of deoxy-, oxy-, and metmyoglobin forms for both WT and mutant are identical (Supplementary Fig. 8). On the contrary, WT and p.His98Tyr feature slightly different reduction potential (*E*°’) values. Spectroelectrochemical investigations yielded a *E*°’ of (+0.040 ± 0.005) V vs SHE for WT MB, in excellent agreement with previous findings^24^, whereas the reduction potential of the p.His98Tyr myoglobin is shifted toward lower values (Fig. 6). *E*°’ determines the tendency of electron transfer from the Fe (II) center to the bound dioxygen ligand^25^, a critical step of the autoxidation process yielding inactive MetMB. From a purely thermodynamic point of view, lower *E*°’ values would favor formation of auto-oxidized MetMB.

**Fig. 7:**
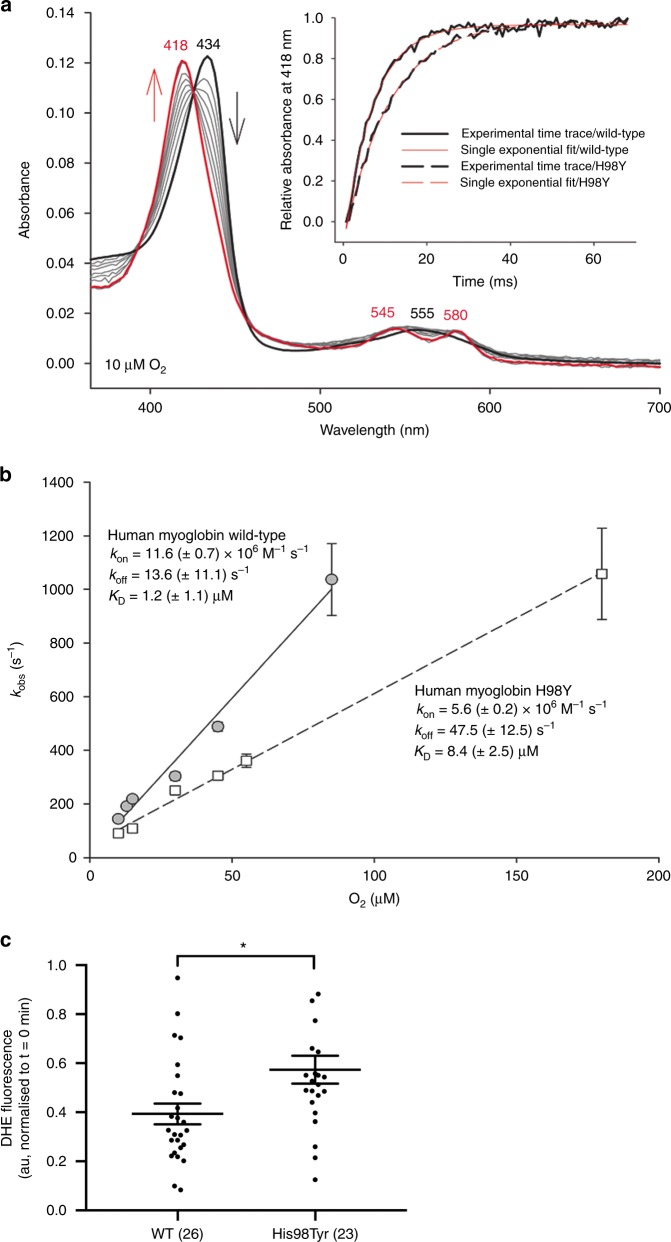
Kinetics of dioxygen binding to wild-type human myoglobin and the variant His98Tyr. **a** Spectral changes upon reaction of 1 µm ferrous wild-type hMb (black line) with 10 µm O_2_. The final spectrum represents oxymyoglobin (red line, 68 ms after mixing). Gray lines represent spectra obtained at 0.68, 2.72, 4.08, 6.12, 8.84, 12.24, 34.00, and 51.00 ms after mixing. The inset depicts experimental time traces at 418 nm of wild-type hMb (solid black line) and p.His98Tyr MB (dashed black line) mixed with 10 µm O_2_ and corresponding single-exponential fits (solid red line, wild-type MB; dashed red line, His98Tyr MB). **b** Linear dependence of *k*_obs_ values from the O_2_ concentration for wild-type MM (gray circles, solid line) and p.His98Tyr MB (white squares, dashed line). **c** Basal intracellular superoxide levels in HEK293FT cells expressing WT or mutant MB-EGFP. Data presented as individual data points and the mean ± SEM, numbers in parenthesis represent *n*. *indicates *p* = 0.007 (Mann–Whitney test, two-tailed)

Another important difference between WT and mutant MB concerns the affinity for molecular oxygen. Oxygen-binding studies to both WT and mutant deoxymyoglobins showed formation of oxymyglobin states (Soret band at 418 nm and two distinct bands at 545 and 580 nm) (Fig. 7). Calculation of rate constants, *k*_on_, of O_2_ binding from the slope of the linear plots of *k*_obs_ values versus O_2_ concentration (Fig. 7b) yielded apparent bimolecular binding rates of (1.16 ± 0.7) × 10^7^ M^−1^ s^−1^ (WT MB) and (5.6 ± 0.2) × 10^6^ M^−1^ s^−1^ (p.His98Tyr), respectively. From the intercept, *k*_off_, of these plots, the dissociation constants, *K*_D_ (=*k*_off_/*k*_on_), were calculated to be (1.2 ± 1.1) µm and (8.4 ± 2.5) µm, respectively (see Table 2), demonstrating a reduced affinity of the mutant MB for molecular oxygen.

To shed light onto the structural and dynamic changes caused by the His-to-Tyr substitution underlying the complex biochemical picture described above, we performed molecular dynamics (MD) simulations of both WT and mutant species. The analysis of our sampling suggests that the substitution does not lead to major global structural rearrangements. The global fold of the protein appears to be maintained (Fig. 6), and the two proteins feature almost superimposable fluctuation patterns (Supplementary Note 2 and Supplementary Figs. 9 and 10). Nevertheless, MD simulations indicated that the heme group in p.His98Tyr is more solvent exposed than in the WT protein. This may explain the shift of *E*°’, which is highly (though not solely) influenced by the extent of exposure of the heme to solvent^26,27^; the latter might also contribute to the increment of *K*_D_ for O_2_ binding to p.His98Tyr, owing to the more polar environment resulting from enhanced accessibility to water.

 Initial studies using a reporter cell assay, suggest that mutant myoglobin may result in elevated intracellular superoxide levels. HEK cells transfected with MB^p.His98Tyr^-EGFP had significantly elevated levels (1.45-fold, *p* = 0.007) of intracellular superoxide compared with those transfected with wild-type MB-EGFP (Fig. 7c). Further experimental investigations of structural and functional changes caused by the substitution will shed more light on the altered relationship between structure, dynamics, and function in the p.His98Tyr variant.

## Discussion

Myoglobin belongs to the globin superfamily. Mutations in hemoglobin have been recognized for a long time and result in hemoglobinopathies^28^. Two other globin proteins (cytoglobin and neuroglobin) have not yet been associated with disease^29^. Our findings allow us to describe the first disorder caused by a mutation in *MB*. We propose the disorder should be named Myoglobinopathy. Family 3, first described by Edstrom et al.^30,31^in 1980, became a classic of the muscle disease literature but its cause remained unsolved until now. Myoglobinopathy has a recognizable clinical phenotype, characterized by disease onset during the fourth or fifth decade of life, manifesting with proximal weakness in the lower extremities and axial muscles, progressing to involve proximal and distal muscles of all limbs with later generalized weakness. Although initially described as a distal myopathy^30^ our study demonstrates that all patients exhibit proximal weakness as the initial symptom. Respiratory insufficiency is a frequent complication and is the cause of death in the majority of patients. Indeed, most advanced patients will require non-invasive respiratory support. Cardiomyopathy was demonstrated in less than half of the patients. It is uncertain whether myocardial involvement is an inconstant feature of the disease, or it remains subclinical for a long time. In support of this last hypothesis, post mortem examination of cardiac tissue in two individuals who did not manifest symptoms of cardiac failure showed large numbers of sarcoplasmic bodies.

The combination of proximal and axial weakness and respiratory insufficiency share some similarities with the phenotype of patients suffering from late-onset Pompe disease. However, cardiomyopathy is usually not a feature of late onset Pompe disease; moreover, distal muscle involvement as revealed by clinical examination and muscle imaging studies does not occur in late onset Pompe diseases until very late in the course of the illness^32^.

The sarcoplasmic bodies are the pathological hallmark of the disease and are the principal aid to diagnosis. They have some similarities with lipofuscin^33^ and also with the globular dense inclusions found in Pompe disease^33,34^ but they are much denser and homogeneous, and usually lack the vacuolar lipid droplets seen in these two conditions. They are also highly reminiscent of the inclusions found in the skeletal muscles from patients with chronic vitamin E deficiency, which are known to be the result of lipid oxidation as a consequence of oxidative stress^35^.

MB accomplishes its physiological tasks thanks to an extremely delicate balance of efficient oxygen binding, resistance to autoxidation, and high heme affinity. The full interplay between the molecular properties affected by the pathogenic mutation is obviously complex. However, it is apparent from our biochemical characterization that some key properties are altered upon mutation, each with potential repercussions at the physiological level. The increased *k*_-H_ would make the mutant more prone to heme loss, as previously seen with variant sperm whale myoglobin^21,22^ and this could contribute to the formation of the lipid and/or protein aggregates found in patients’ muscle owing to the generation of ROS by free heme^36^. In support of this hypothesis, we have shown experimentally, via a reporter cell assay, that mutant myoglobin appears to be associated with elevated levels of intracellular superoxide. As a consequence, although myoglobin is expressed in oxidative myofibers, the impact of the mutation may be reflected in both myofiber types. The *E°’* shift may impact on myoglobin’s role as a regulator of redox pathways in muscle, for example unbalancing the redox-dependent mechanisms of oxygen-controlled NO formation^37–39^, whereas the higher *K*_D_ of the mutant MB for O_2_ might affect the capability of MB to act as an oxygen storage species.

To date, all myoglobinopathy cases have been caused by the same c.292C>T, (p.His98Tyr) missense variant in *MB*, thus suggesting that this position may be a mutational hotspot. We demonstrate that the c.292C>T, (p.His98Tyr) missense variant is associated with altered oxygen binding and increased tendency to heme loss. It remains to be seen whether other variants in *MB* result in the same disease.

## Methods

### Patients

The 14 patients described in this study constitute a clinically and pathologically homogeneous cohort of adult patients, belonging to six unrelated families (F1–F6), (Fig. 1), originating from Spain (2), Sweden (1), France (2), and The Netherlands (1). The families were principally identified by their highly characteristic features on muscle biopsies. The clinical and pathological features of the Swedish family have been described^34,35^ and the Dutch patient was briefly described in a workshop report^40^.

The study was approved by the ethics committees of the participating institutions: IDIBELL-Hospital de Bellvitge, Karolinska Institutet, University of Western Australia, Institut de Myologie, GHU Pitié-Salpêtrière, Hospices Civils de Lyon, University Hospitals Leuven and Henri Mondor University Hospital Sample collection was performed with written informed consent from the patients according to the declaration of Helsinki.

### Muscle biopsy

A muscle biopsy was performed for at least one affected patient per family after written informed consent (Supplementary Table 1). In addition, post mortem cardiac, skeletal, and diaphragm muscle samples were obtained from individuals F1, II: 7 and post mortem cardiac and skeletal muscle was obtained from individual F3: III: 5. Samples were processed for routine histochemical analysis and for acid phosphatase activity, and Perls’ Prussian blue stain and Alizarin Red for iron and calcium detection, respectively. Immunohistochemical analysis was performed for myoglobin, ubiquitin, p62, and LAMP1. Cryostat sections, 8 μm thick, were incubated with 1% hydrogen peroxide in 1 × PBS (Phosphate Buffer Saline) for 5 min in order to block endogenous peroxidase. Then, unspecific binding reactions were blocked with 10% (goat) or 3% (horse) normal serum in 1 × PBS for 2h, followed by incubation of the corresponding primary antibody diluted in 1 × PBS with 3% (goat) or 1% (horse) normal serum at 4 °C overnight. Mouse monoclonal anti-myoglobin (Novus biologicals), anti-LAMP1 (Santa Cruz Biotechnology, H-228:sc-5570), rabbit polyclonal anti-ubiquitin (Dako, z-458), and guinea pig polyclonal antibody against the C-terminus of p62 (Progen, GP62-C) were used at a dilution of 1:50, 1:100, 1:100, and 1:100, respectively. After washing, the sections were processed with Super Sensitive Link-Label IHC Detection System (BioGenex) following the instructions of the manufacturer. As sarcoplasmic bodies are brown and diaminobenzidine (DAB) immunoreactions appear brown, the immunoreactions were visualized as a dark blue precipitate using NH_4_NiSO_4_ diluted in phosphate buffer with 0.03% DAB, 0.04% NH_4_Cl, and 0.001% hydrogen peroxide^41^. Sections were viewed with an Olympus BX43 microscope. Non-stained sections were also examined with a Leica TCS-SL confocal microscope under a wide range of visible laser excitation lines. A small sample of biopsy tissue was fixed in 2% glutaraldehyde, postfixed with 1% osmium tetroxide, and embedded in araldite. Ultrathin sections were stained with uranyl acetate and lead citrate, viewed with a JEOL 1011 electron microscope and photographs taken with a gatan 782 camera.

### Molecular genetics—the two Spanish families

Targeted capture and next generation sequencing (ProtonTM, Life Technologies) of 336 genes including 254 neuromuscular disease genes listed in the Neuromuscular Disorders Gene Table at the end of December 2012 (www.musclegenetable.fr) was performed in probands from families F1 and F2. Exome enrichment was performed on DNA from the proband (using an Ampliseq Whole Exome kit, Thermofisher Scientific). In brief, a total of 100 ng of DNA was amplified in 12 separate PCR pools, each containing ~ 25,000 primer pairs under the following conditions: 99 °C for 2 min followed by 10 cycles of 99 °C for 15 s and 60 °C for 16 min. After amplification, the individual reactions were pooled and digested using the supplied FuPa enzyme that degrades the PCR primers. Next, barcoded sequencing adaptors were ligated and the library was purified using AMPure beads (Beckman Coulter) and amplified for five cycles using Platinum high-fidelity polymerase. The final amplified library was purified again using AMPure beads and analyzed on a 2100 Bioanalyser (Agilent Technologies). Libraries were diluted to 18–26 pM. Attachment to Ion Sphere Particles was done with the Ion Proton Template 200 V3 kit. Sequencing was performed on P1 sequencing chips (520 flows) using an Ion Proton sequencer and Ion Sequencing 200 kit V3. After pooling, two samples were sequenced on one chip. After sequencing, to remove low-quality bases from the 3’ end, reads were trimmed. Reads were mapped to the genome reference sequence (Hg19) using tmap (Torrent Suite 4.2). Torrent Variant caller was used to call variants with exome-optimized custom settings. Ion Reporter 4.0 was used to annotate the data. Filtering of variants was performed with ANNOVAR against three databases: ENCODE GENECODE v.19, 1000genomes (threshold > 0.5%), dbSNP138 and also against a list of common in-house variants^42^. The filtered exome data were interrogated for variants in a set of genes that were most highly enriched (>1000-fold) in skeletal muscle samples within the FANTOM5 (functional annotation of the mammalian genome) data set^8^. Using the FANTOM5 promoter level expression atlas, we generated a ranked list of skeletal muscle enriched genes. Source file with pre-computed relative expression was downloaded from http://fantom.gsc.riken.jp/5/tet/data/hg19.cage_peak_phase1and2combined_rel_expr.txt.gz. We identified the promoter for every gene with the most enriched expression in 76 skeletal muscle samples and compared this with the median expression across the entire FANTOM5 collection [log10(max expression in any skeletal muscle sample + 1)—log10(median expression in the FANTOM5 collection + 1)]. A promoter for 82 annotated protein-coding genes was identified showing > 1000-fold enriched expression in skeletal muscle. Thirty-one of these genes had previously been associated with a genetic muscle disease in humans. The 51 genes that had not previously been associated with human muscle disease we prioritized as candidate myopathy genes.

### Genome-wide linkage analysis of the Swedish family—Family 3

Genome-wide linkage analysis was performed using genotypes of 550 microsatellite markers. Genotype errors were checked and removed prior to analyses by using PedCheck^43^ GENEHUNTER v. 2.1^44^ as used for both parametric and non-parametric linkage analysis. Parametric linkage analysis was performed assuming an autosomal dominant model with a penetrance of 0.999 for heterozygotes, disease-allele frequency of 0.001, and phenocopy rate of 0.001. LOD scores were calculated under the same dominant model. Both multipoint and single-point analyses were performed. The positions of marker loci were based on published human genetic maps (National Center for Biotechnology Information).

### Target sequencing in the Swedish Family

Targeted capture and sequencing included > 400 genes surrounding the peak multipoint LOD score. Enrichment for the target-sequencing region was obtained by using a custom-designed NimbleGen SeqCap EZ Developer library (Roche NimbleGen, Inc. Madison, WI 53719 USA). Eight samples, two healthy controls, and six patients, from the Swedish family, F3, were selected for targeted sequencing. The sequencing was done at SciLifeLab Stockholm, Sweden. All samples yielded the expected number of sequencing reads. Each DNA library was prepared from 3 μg of the pooled genomic DNA. DNA was sheared to 300 bp using a Covaris S2 instrument and enriched by using the custom-designed NimbleGen SeqCap kit. The clustering was performed on a cBot cluster generation system using a HiSeq paired-end read cluster generation kit according to the manufacturer’s instructions. The samples were sequenced on an Illumina HiSeq 2000 as paired-end reads to 100 bp. The sequencing runs were performed according to the manufacturer’s instructions. Base conversion was done using Illumina’s OLB v1.9.

### Sanger sequencing

The *MB* c.292C>T (p.His98Tyr) variant was identified in Families F1 to F3 through NGS. The presence of the variant in these three families was Sanger confirmed. Families F4, F5 and F6 were screened for mutations in *MB* using Sanger sequencing. Segregation studies were performed in all families including samples from all available affected and unaffected relatives using Sanger sequencing. MB primers are included in Supplementary Methods 1.

### In silico predictions

The in silico pathogenicity predictors used were: MutationAssessor^45^, MutationTaster^46^, PolyPhen-2^47^, Provean^48^, and SIFT^49^.

### Haplotype analysis

To test for possible founder effects, we studied eight informative microsatellite markers (D22S685, D22S691, D22S1152, D22S1265, (*MB*), D22S277, D22S683, D22S692, IL2RB) spanning 3 Mb around *MB* using multiplex polymerase chain reaction with labeled primers and fragment analysis. GeneMapper software was used for the fragment analysis. The primers used in haplotype analysis are included in the Supplementary Methods 2.

### Correlative electron microscopy and NanoSIMS imaging

For NanoSIMS (Nanoscale Secondary Ion Mass Spectrometry) imaging, 500 nm sections of resin embedded muscle samples from individuals F1 II: 5, and II: 7 and F2 II:2 were mounted on platinum coated coverslips, and then transferred into a FEI Verios scanning electron microscope (SEM) for backscattered electron (BSE) imaging^50^. A 1–2 kV electron beam with current of ~ 100pA was used for BSE imaging. Sections were then coated with 5 nm platinum by a SC 7640 Polaron sputter coater, and transferred into the NanoSIMS 50L (CAMECA, France) for chemical analysis. The same areas imaged by SEM were analyzed by the NanoSIMS for chemical distribution. A 16 keV Cs^+^ primary beam is used to detect ^31^P^–^, ^32^S^–^, and ^56^Fe^16^O.

For comparative purposes, in addition to myoglobinopathy samples, we performed NanoSIMS analysis in one sample from a patient with Pompe disease containing electron-dense inclusions, two samples showing abundant lipofuscin and two muscle samples from patients affected with distal myopathies with rimmed vacuoles.

### Fourier transform infrared microscopy

10 μm-thick cryostat muscle sections were obtained from individuals F1, II: 5 and II: 7, F2, II:2, F3, III:5, II:15 and IV:6 and deposited on CaF_2_ windows and allowed to dry. Samples were kept at vacuum conditions before the infrared spectroscopy analysis. μFTIR experiments were performed at the MIRAS beamline in the ALBA synchrotron, Spain using a Hyperion 3000 Microscope coupled to a Vertex 80 spectrometer (Brucker) equipped with 36x magnification objective. The measuring range was 900−4000 cm^−1^ and the spectra collection was done in transmission mode at 4 cm^−1^ spectral resolution, 10 μm × 10 μm aperture dimensions using an mercury cadmium telluride (MCT) detector. For each spectrum 128 scans were co-added. Background spectra were collected from a clean area of each CaF_2_ window. FTIR data were analyzed using OPUS 7.5 (Brucker) and Origin 9.1. The spectra exhibiting strong Mie scattering were eliminated and second derivation of the spectra was applied in order to eliminate the baseline and improve the resolution of the different bands using a Savitzky−Golay algorithm with a 15-point filter and a polynomial order of two. The assignments of Infrared bands are well-established for proteins and lipids and are shown in Fig. 5c. The derivative of each FTIR spectrum was computed and the ratios for lipid oxidation (1741 cm^−1^/2925 cm^−1^, CO/−CH_2_) and lipid protein ratio (2925 cm^−1^/1654 cm^−1^ Amide I/−CH_2_) calculated^51^.

### Expression and purification of recombinant WT and mutant MB

pET-19b plasmids with human WT, p.His98Tyr or p.His65Tyr/Val69Phe myoglobin cDNA (the latter to be used for heme loss kinetic studies, see below) cloned into them were purchased (GenScript USA Inc.) and then transformed in *Escherichia Coli* BL21(DE3) for the production of N-terminally His-tagged fusion myoglobins. Luria-Bertani medium supplemented with ampicillin (100 μg/ml) was inoculated with a freshly prepared overnight culture (at a dilution ratio of 1:100). The culture was grown at 37 °C under agitation (220 rpm) until OD_600_ = 0.6 was reached. The temperature and the shaker speed were reduced to 20 °C and 100 rpm and hemin was added to a final concentration of 15 μg/mL. After 30 min, isopropyl-β-d-thiogalactopyranoside was added to a final concentration of 0.5 mm. Please note that p.His65Tyr/Val69Phe for the hemin loss rate experiments was grown using the same procedure but no hemin was added. To make sure that the double mutant was in the apo form, 3.5 mg of protein at pH 2.5 were extracted using an equal volume of 2-butanone, shaking three times for 5 min and then incubating the mixture for 10 min at 4 °C^20^. The aqueous phase was dialyzed at 4 °C against 1 l of water and then against 1 l of 0.2 m phosphate buffer pH 7 or 0.2 m acetate buffer pH 5. After 12 h, the culture was centrifuged, resuspended in 50 mm phosphate buffer, 150 mm NaCl, and 20 mm imidazole (pH 7.5), lysed by sonication and clarified by centrifugation. The proteins were purified by affinity chromatography (HisTrap FF crude columns (GE Healthcare) connected to an AKTA Prime chromatographic system (Amersham Pharmacia Biotech) and subsequently by gel filtration chromatography (HiLoad 16/60 Superdex 75 prep grade (Amersham Biosciences). The purity of the fractions was checked by sodium dodecyl sulfate polyacrylamide gel electrophoresis and UV-Vis spectroscopy.

### WT and His98Tyr variant myoglobin aggregation test

WT MB and the variant His98Tyr MB were incubated in 10 mm phosphate buffer at pH 7.4 at 37 °C and 200 rpm agitated for 1 week. At different times, aliquots were taken for ThT Fluorescence and Infrared Spectroscopy measurements. For fibril fluorescence detection, Thioflavin T (ThT) (SIGMA-Aldrich), a fluorescence dye specific to fibrils, was added to the solution. In total, 8 mm ThT stock solutions were prepared in 10 mm phosphate buffer at pH 7.4 buffer and filtered through a 0.45 μm syringe driven filter unit (Millipore). ThT final concentration in the fluorescence cuvette was 35 μm. Excitation and emission wavelengths were set at 450 and 490 nm, respectively, using a PTI fluorimeter. Temperature was 37 °C. For Infrared spectra, 3 μL of the different samples were deposited on CaF_2_ windows and dried under vacuum. Infrared spectra were measured at MIRAS beamline in ALBA synchrotron.

### Electrochemical measurements

All spectroelectrochemical experiments were conducted in a homemade OTTLE (optical transparent thin-layer spectroelectrochemical) cell, in a three-electrode configuration that consisted of a gold minigrid working electrode (Buckbee-Mears, Chicago, IL), an Ag/AgCl/3M KCl microreference electrode (373/SSG/6, Amel electrochemistry, Milano, Italy) and a platinum wire as the counter electrode. Potentials were applied across the OTTLE cell with an Amel model 2053 potentiostat/galvanostat. A constant temperature was maintained by a circulating water bath, and the OTTLE cell temperature was monitored with a micro thermo-couple. UV−vis spectra were recorded using a Varian Cary C50 spectrophotometer. The OTTLE cell was flushed with argon gas to establish an oxygen-free environment in the cell. Experiments were conducted at a constant temperature of 25 °C, using 600 μL samples containing 15 μm of protein in 50 mm phosphate buffer (pH 7.0) 20 mm NaCl and 30 μm phenazine methosulfate. Nernst plots consisted of at least five points and were invariably linear with a slope consistent with a one-electron reduction process. Square wave voltammograms were recorded using a Potentiostat/Galvanostat mod. 273A (EG&G PAR, Oak Ridge, USA). A graphite disk electrode, a Pt wire and a saturated calomel electrode (SCE) were used as working, counter and reference electrode, respectively. The electric contact between the SCE and the working solution was achieved with a Vycor® (PAR) set. The working electrode was cleaned before each use with mechanical polishing with 0.5 μm aluminum oxide followed by 5 min in an ultrasound bath. Myoglobin was deposited on the electrode by drop casting 10 μL of a solution containing 100 μm protein and 20 mm phosphate buffer (pH 7.0). The electrode was then incubated overnight at 4 °C. The following step was to drop cast 20 μL of a solution containing 10% m/m of gelatin type A (Sigma-Aldrich, G1890) and 20 mm phosphate buffer (pH 7.0), followed by incubation at room temperature for 2 hours. The electrode was then ready for electrochemical measurements. The working solution was 20 mm phosphate buffer (pH 7.0). Experiments were carried out under argon atmosphere at 25 °C. The errors associated to the E°’ values are the standard deviations calculated over at least three independent sets of measurements for each species.

### Measurement of hemin loss rates for WT and p.His98Tyr

Hemin dissociation kinetics was investigated as follows: we monitored the decrease with time (0–350 min) of the absorbance at 410 nm for the transfer of the heme from holo WT (or p. His98Tyr) MB to the apo MB double mutant p.His65Tyr/Val69Phe^20,21^ in a 1:10 concentration ratio. Time courses were fitted to a single-exponential expression in GraphPad Prism 5.0 (GraphPad Software, Inc., San Diego, CA) to obtain the first order dissociation rate constant for hemin loss *k*_-H._ The experiments were conducted at 37 °C in the presence of 0.45 m sucrose, in 0.2 m phosphate buffer at pH 7 or 0.2 m acetate buffer pH 5. UV−vis spectra were recorded using a Varian Cary C50 spectrophotometer.

### Molecular modeling

For each protein, three 350 ns-long MD simulations were produced using the 5.0.4 version of the GROMACS software. The starting structure of human myoglobin was taken from the PDB file 3RGK^52^. The program Modeller^53–56^ was used to edit the 3RKG.pdb file. The structure of the C-terminus was taken from the PDB file 1MWD^57^. The structure of the p.His98Tyr mutant was obtained by homology modeling using Modeller and taking the 3RGK file as template structure. All the simulations were performed in the reduced ensemble. The starting structures were first relaxed in vacuum using the steepest descent algorithm, 3000 steps, and then put in a rhombic dodecahedral box, filled with TIP3P water molecules. Na^+^ and Cl^−^ were added to neutralize the protein total charge and to have a final concentration of 100 mm. The systems were then relaxed with the conjugate gradients algorithm in two steps. Then the systems were annealed to 300 K, starting from 50 K, in a 100 ps run. The systems were then equilibrated in three successive runs, each 500 ps long: (i) an NVT at 300 K^58^, (ii) an NPT at 300 K, 1 bar (iii) an NPT at 300 K and 1 bar^59^. The last frame was used as a starting structure for the MD production run in the NPT ensemble, at a temperature of 300 K and a pressure of 1 bar. All bond lengths were constrained using the LINCS algorithm^60^ and an integration step of 2.0 fs was used. Long-range electrostatics was computed by the Particle Mesh Ewald method^61^, with a grid spacing of 0.12 nm combined with a fourth-order cubic interpolation and a real space cutoff of 0.9 nm. During all the runs, the CHARMM27 force field^62^ with CMAP correction^63^ was used. The three replicates for each system were made using the same starting structure, but changing the seed for the velocities random generator. The determination of the Solvent Accessible Surface Area (SASA), the analysis of the distances, the analysis of secondary structure, the Essential Dynamics (ED), the root mean square fluctuations calculations and the clustering analysis, were performed using the corresponding Gromacs packages. The ED analyses were performed using the backbone atoms of residues 1–150 both for the fitting and the calculation of the covariance matrix. The clustering procedure was made with a cutoff of 0.11 nm and the Gromos method^64^. The centroids of the first clusters were used for the structure comparison. ED analysis was used to investigate the conformational space spanned by WT and mutant p.His98Tyr myoglobin within the molecular dynamics (MD) simulation and visualize the principal motions associated with each species. To assess the differences between the fluctuation patterns characteristic of WT myoglobin and the p.His98Tyr mutant, the equilibrated trajectories were linked for the two species into a single molecular dynamics ensemble (concatenated trajectory) and essential dynamics analysis was performed. The concatenated trajectory was projected onto a plane defined by the first two eigenvectors of motions, which are usually referred to as the essential plane. This allowed a two-dimensional representation of the conformational landscape explored during the MD simulations, and for the calculation of the corresponding Free Energy Landscape.

### Oxygen binding to wild-type human myoglobin and the variant His98Tyr

Time-resolved binding of oxygen (O_2_) to wild-type human myoglobin and the variant His98Tyr was monitored by stopped-flow spectroscopy in the conventional mixing mode. The stopped-flow apparatus (model SX 18MV, Applied Photophysics) was equipped with a diode-array detector and an optical quartz cell with a path length of 10 mm (volume: 20 µL). The fastest time of mixing was 0.68 ms. All measurements were performed at 25 °C. In order to establish O_2_-free starting conditions, all solutions were degassed with Argon. The stopped-flow apparatus (all syringes and tubings) was extensively flushed with O_2_-free buffer (20 mm phosphate buffer, pH 7.0; 20 mm NaCl). The concentration of wild-type MB and of the variant in the cell was 1 µm. Prior to measurements, the degassed O_2_-free protein solutions were reduced with sodium dithionite (final concentration 25 µg mL^−1^). To test oxygen binding, O_2_-free buffer was flushed with O_2_ for different time spans and the O_2_ concentration was determined using a thermostatted (25 °C) Clark-type electrode (Oxygraph Plus; Hansatech Instruments, Norfolk, United Kingdom). The electrode was equilibrated to 100% O_2_ saturation by bubbling O_2_ through the buffer and for 0% saturation by bubbling with N_2_ until the respective plateaus were reached to derive an offset and a calibration factor. Experiments were conducted in 20 mm phosphate buffer, pH 7.0, supplemented with 20 mm NaCl. Dioxygen binding was determined by monitoring the absorbance increase at 418 nm. The apparent second-order rate constants, *k*_on_, were obtained from the slope of a plot of *k*_obs_ values (derived from single-exponential fit of the respective time traces at 418 nm) versus the dioxygen concentration; *k*_off_ was derived from the intercept of this plot.

### Assessment of cellular superoxide

HEK293FT cells (p15-20) (Invitrogen) were seeded onto coverglass pre-coated with Matrigel (Corning) in 12-well tissue culture plates. Cells were transfected with pcDNA3.1 expression plasmids containing full length human wild-type (MB^WT^) or c.292C>T *MB* (MB^p.His98Tyr^) cDNA fused with an EGFP tag (Genscript). Transfections were performed using 1 μg of DNA and Lipofectamine® 3000 (Thermofisher) as per manufacturer’s instructions. Experiments were performed 48–72 h post transfection and were conducted over three independent experiments. Plasmids are available upon request. Basal superoxide generation was assessed^65^ in individual cells expressing MB^WT^ and MB^p.His98Tyr^ (as indicated by EGFP positivity) using the fluorescent indicator dihydroethidium (DHE, 5 μm, 515–560 nm ex filter, 590 long pass em) at 37 °C. Fluorescent DHE signal was measured on a Hamamatsu Orca ER digital camera attached to an inverted Nikon TE2000-U microscope. Fluorescent images were taken at 1 min intervals with 200 ms exposure. Metamorph 6.3 was used to quantify the signal by manually tracing individual cells. An equivalent region not containing cells was used as background and was subtracted. DHE fluorescence for each cell was recorded as the slope of the signal measured over the course of 20 min, normalized to fluorescent signal recorded at 1 min.

## Supplementary information

Supplementary Information

Source Data

## Data Availability

The source data underlying Figs. 5, 6, and 7 are provided in the Source Data file. The novel variant here reported has been deposited in ClinVar, accession number: SCV000882438. Patients’ consents were not obtained for public deposition of sequencing data, but these are available upon reasonable request. Any additional data that support the findings of this study are available from the corresponding author upon reasonable request.
