# Supplementary material for: Myoglobinopathy is an adult-onset autosomal dominant myopathy with characteristic sarcoplasmic inclusions
Source: Nat Commun. 2019 Mar 27;10:1396. doi: 10.1038/s41467-019-09111-2 (PMC6437160; doi:10.1038/s41467-019-09111-2)
Supplement: Supplementary file 1 — Supplementary Information [file 41467_2019_9111_MOESM1_ESM.pdf]

Supplementary Information

Myoglobinopathy is an adult-onset autosomal dominant myopathy with characteristic sarcoplasmic inclusions

Olivé M, *et al.*

1. Supplementary methods
2. Supplementary notes
3. Supplementary tables
4. Supplementary figures

1. Supplementary methods

Supplementary methods 1

MB primers

MB exon 1 Forward	GCCATTGAGCGATCTTTG
MB exon 1 Reverse	GATTCTCATGCTTCCTCCAG
MB exon 2 Forward	GAAGTGAATGGCCCAAG
MB exon 2 Reverse	TACCCATGGAGAGGATGC
MB exon 3 Forward	CTCAATACACGGTCCTGGA
MB exon 3 Reverse	ACAAAGCAGACACTCAGAAGC

Supplementary methods 2

Primers used for the haplotype analysis using microsatellite markers

D22S685_fwd	TTCTTAGTGGGGAAGGGATC
D22S685_rev	TGAGTTTGATGTTTTTGATAGACA
D22S691_fwd	TCAAATTGGAGCCTCTTCTG
D22S691_rev	GCTCATGTCTCCAAATGGAC
D22S1152_fwd	CTCAGGGTGCCTTGGAAT
D22S1152_rev	TGGGTCCTCTCAAAGCAAA
D22S1265_fwd	CAGGACAGTTCATGGAGCTT
D22S1265_rev	ATCCTTAAAGGCTCGGCTTA

D22S277_fwd	TTCTTGTGTGGTAGTCTGGG
D22S277_rev	TACCNACTCCCCAAACTATG
D22S683_fwd	AACAAAACAAAACAAAACAAACA
D22S683_rev	GGTGGAAATGCCTCATGTAG
D22S692_fwd	AAGCTATGATCACGTCACTGC
D22S692_rev	GGTGACTAGCCAATATCCTCC
IL2RB_fwd	GCTAGATTTTCCCCGATGAT
IL2RB_rev	ATGTAAAGTGCTCTCAAGAGTGC

2. Supplementary notes

Supplementary note 1

MB aggregation test

Following the identification of β-sheet structures in some sarcoplasmic bodies, we wanted to further investigate the capacity of the mutant MB to form amyloid fibrils *in vitro* by using infrared spectroscopy in combination with Thioflavin (ThT) fluorescence. Results of our analysis demonstrated that both the WT and mutant His98Tyr MB have some capacity to form β-sheet protein aggregates. This was demonstrated after the observation of an increased intensity at 1627 cm^{-1} (typical of intermolecular β-sheet structures) (Supplementary Fig. 6) after 24h of MB incubation at 37°C and 200 rpm. However, the ThT measurements did not show any fluorescence increase during the monitored period (8 days) (data not shown), thus proving the absence of fibril formation under the used conditions.

Supplementary note 2

Molecular dynamics simulations

We calculated the root mean square fluctuations (RMSF) per Cα atoms of both WT and p.His98Tyr mutant; the overlay of the corresponding RMSF profile is displayed in Supplementary Fig. 9. The

RMSFs represent the standard deviation over time of the atomic positions with respect to the average structure within our sampling: therefore, they are a measure of the average atomic mobility. Almost superimposable RMSF profiles, as in Supplementary Fig. 9, provide a first evidence of very similar dynamic features for the two species.

We next performed Essential Dynamics (ED) analysis, that allows for a thorough investigation of the conformational space spanned by a protein within a molecular dynamics simulation to characterize and visualize the principal motions associated to a species. As we are mostly interested in *differences* between the fluctuation patterns characterizing WT and p.His98Tyr mutant, we linked the equilibrated trajectories for the two species into a single MD ensemble (concatenated trajectory), performing ED analysis on it. We then projected the concatenated trajectory onto a plane defined by the first two eigenvectors of motions, which is usually referred to as the essential plane. This allows for a two-dimensional representation of the conformational landscape explored during the MD simulations, and for the calculation of the corresponding Free Energy Landscape (FEL). The result is displayed in Supplementary Fig. 10. Although displaying the FEL for both WT and p.His98Tyr, the contour plot shows a single minimum: this is a clear indication that the two proteins explore almost superimposable conformational spaces. Together, data displayed in Supplementary Figure 9 and 10, suggest that the protein dynamic is almost unaltered upon the substitution.

3. Supplementary tables

Supplementary Table 1: Summary of myopathological features in myoglobinopathy

Family/ Individual	Site of biopsy	Years since disease onset	Biopsy from a clinically affected muscle	Sarcoplasmic bodies	Fiber size variation	Internal nuclei	Fibro-fatty tissue proliferation	Vacuoles	Type I fiber predominance
**F1**									
II:5	Deltoid	15	yes	yes	yes	yes	yes	yes	Yes
II:7	Biceps	10	yes	yes	yes	yes	no	yes	Yes
**F2**									
II:2	Biceps	30	yes	yes	yes	yes	no	yes	Yes
**F3**									
III:5	Ant tibialis Quadriceps	8	yes	yes	Yes	yes	yes	yes	no
III:9	Ant tibialis	2	yes	yes	Yes	yes	yes	no	no
III:14	Ant tibialis	-3 *	no	yes	Yes	yes	no	no	no
III:15	Ant tibialis	-10 *	no	yes	No	no	no	no	no
IV:2	Ant tibialis	-3 *	no	yes	No	no	no	no	no
IV:6	Ant tibialis	3	no	yes	No	yes	no	no	yes
**F4**									
II: 6	Deltoid	10	yes	yes	Yes	yes	no	yes	yes
**F 5**									
II: 4	Deltoid	10	yes	yes	Yes	yes	no	yes	yes
**F 6**									
II: 4	Ant tibialis	3	no	yes	Yes	yes	no	yes	yes
II:4	Quadriceps	6	yes	yes	Yes	yes	yes	yes	no
II:4	Gastrocnemius	9	yes	yes	Yes	yes	no	yes	yes

*Minus sign before number indicates years before the onset of symptoms

Supplementary Table 2: Haplotypes or alleles of the six families.

						Family 5 three sibs							
Microsatellite	Hg19 position	Family 1*	Family 2	Family 3	Family 4	1		2		3		Family 6	
D22685	34,595,593	302	302	310	306	306	306	306	306	306	306	314	314
D22S691	34,875,643	222	242/246	223	255	246	242	246	242	246	242	247	223
D22S1152	35,139,045	263	263	263	263	263	263	263	263	263	263	263	269
D22S1265	35,389,908	176	176	176	206	200	191	200	191	200	191	176	188
MB	36,002,811												
D22S277	36,271,500	162/166	162/166	156	150	160	160	160	160	160	160	166	158
D22S683	36,513,691	246	216	226	222	216	246	216	246	216	246	224	230
D22S692	37,125,545	156	160	160	152	160	160	160	160	160	160	160	156
IL2RB	37,536,285	245	251/255	257	245	259	255	259	255	259	255	255	245

*For families 1-4 the haplotype is the haplotype segregating with the disease in the Family. For Families 5 and 6 the alleles in the affected individuals are shown. Double alleles eg 242/246 indicate uninformative markers

4. Supplementary figures

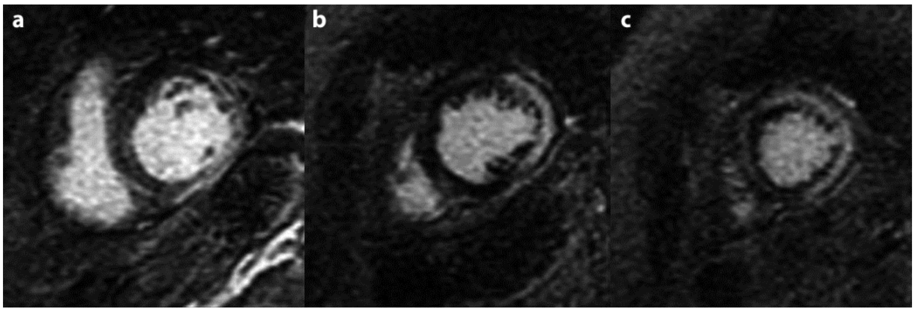

Supplementary Figure 1: Cardiac MRI from patient F2, II: 2. Short axis images after gadolinium administration showing late transmural enhancement in basal and mid inferolateral segments (a, b) and in all of them at the apical level (c), indicative of fibrosis.

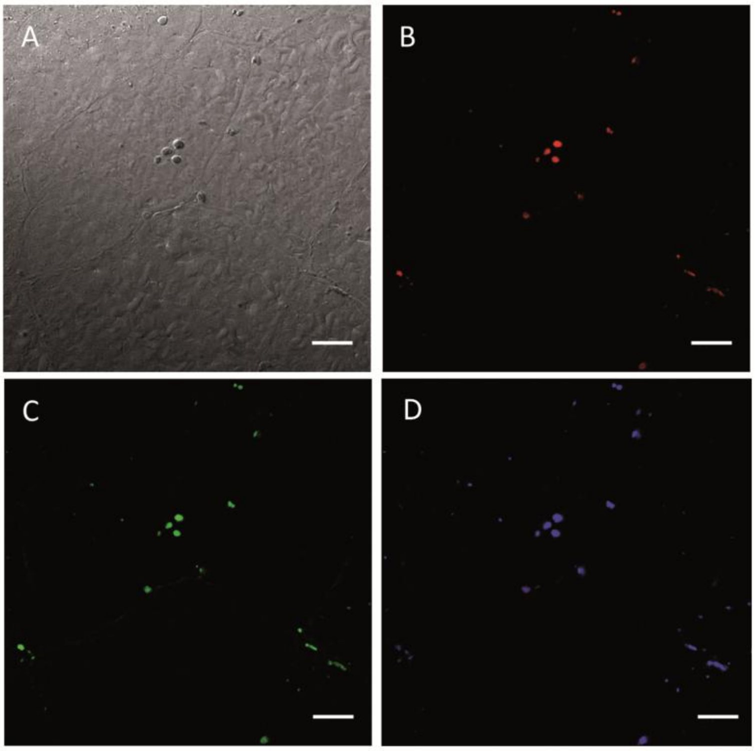

Supplementary Figure 2**:** Autofluorescent aggregates on muscle biopsy. Unstained cryostat sections of skeletal muscle from patient F2, II: 2 viewed under confocal microscopy. The sarcoplasmic bodies exhibit autofluorescence emission in a wide range

of visible laser excitation lines: 488nm (b), 543nm (c) and 633nm (d). Scale bars = 20 µm.

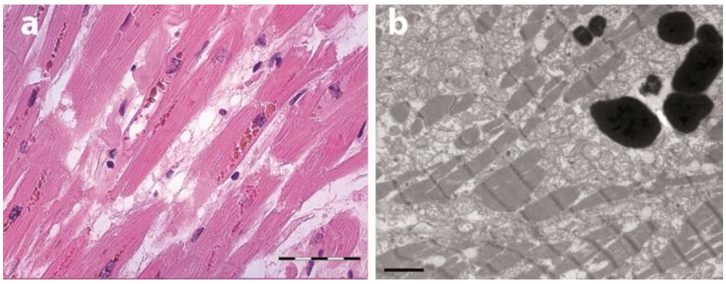

Supplementary Figure 3: Post-mortem cardiac pathological findings. Post-mortem analysis of myocardium from patient F1, VII showing large numbers of sarcoplasmic bodies in cardiac muscle. (a) HE; (b): electron microscopy. Scale bar in a = 20 µm, b = 2 µm.

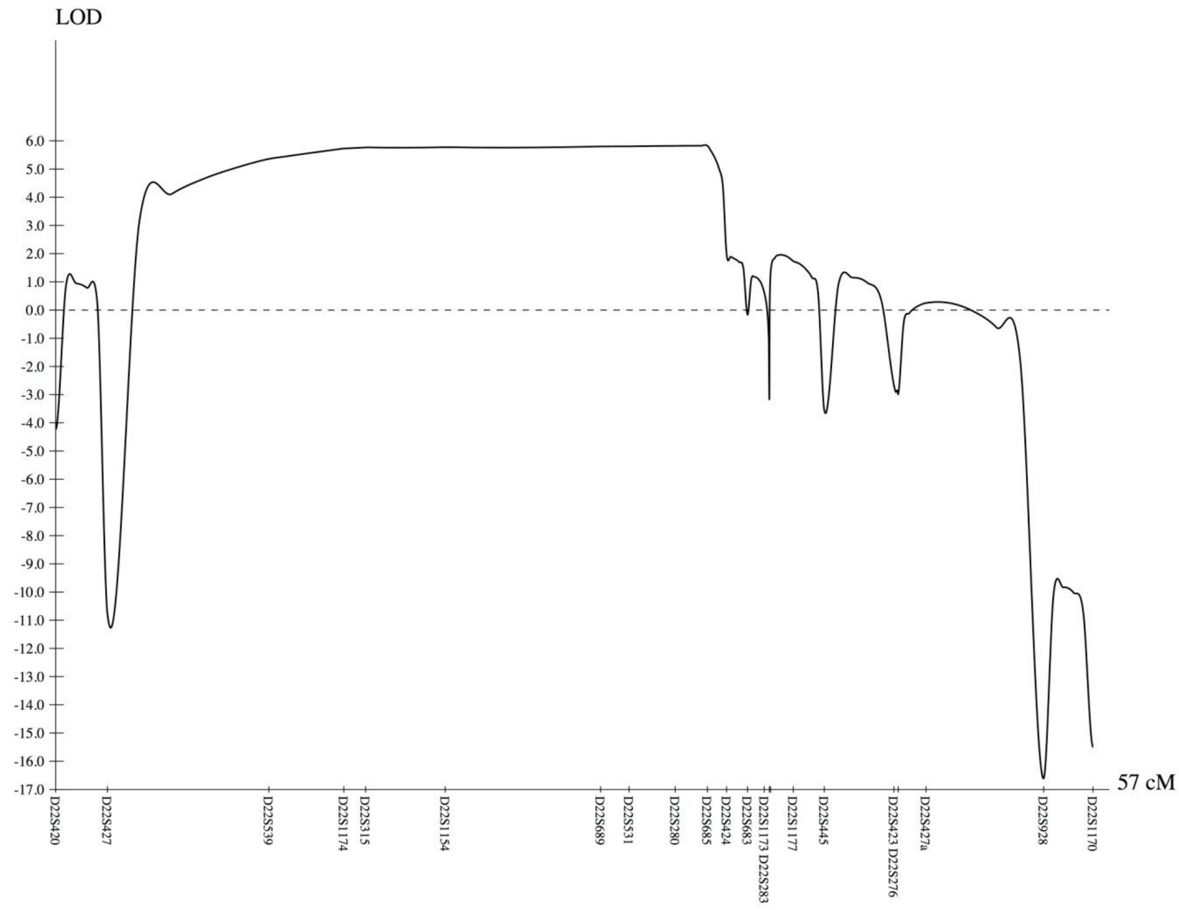

Supplementary Figure 4: Multipoint LOD score plot of the linkage region identified on chromosome 22 for Family 3.

H. sapiens	98	IKPLAQSHATK**H**KIPVKYLEFIS
Mutant	98	IKPLAQSHATK**Y**KIPVKYLEFIS
P. troglodytes	98	IKPLAQSHATK**H**KIPVKYLEFIS
M. mulatta	98	IKPLAQSHATK**H**KIPVKYLEFIS
F. catus	98	IKPLAQSHATK**H**KIPVKYLEFIS
M. musculus	98	IKPLAQSHATK**H**KIPVKYLEFIS
G. gallus	98	IKPLAQSHATK**H**KIPVKYLEFIS
D. rerio	98	IKPLAQSHATK**H**KIPVKYLEFIS

Supplementary Figure 5: The p.His98Tyr variant involves a residue conserved to zebrafish.

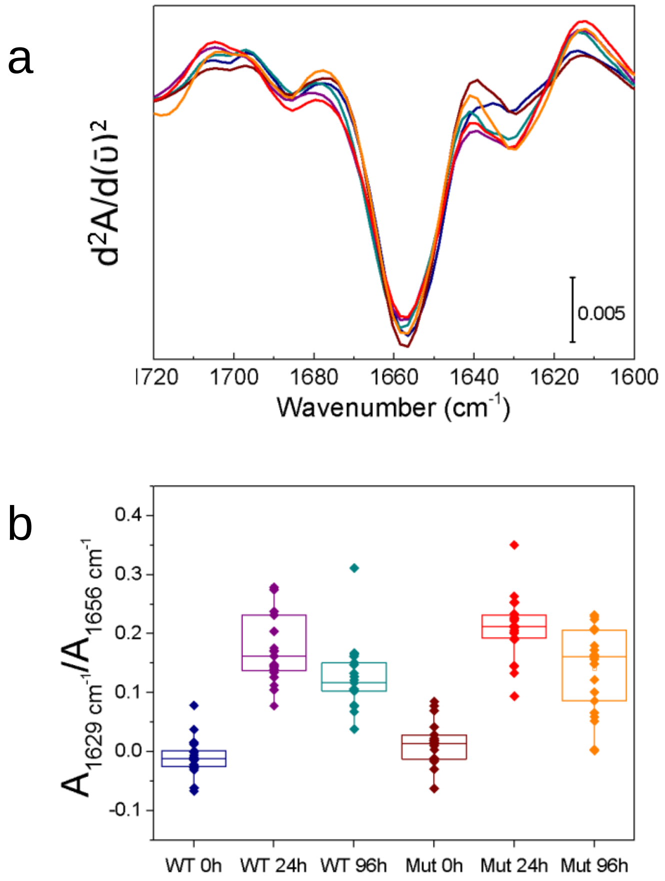

Supplementary Figure 6: WT and His98Tyr MB variant *in vitro* aggregation test. (a) Second derivative spectra of MB-WT and MB-His98Tyr after different incubation times: MB-WT at 0 h in dark blue, MB-WT after 24 h in dark purple and MB-WT after 96 h in dark cian. MB-His98Tyr at 0 h in wine, MB-His98Tyr after 24 h in red and MB-His98Tyr after 96 h in orange. (b) β-sheet aggregation ratio (A1629 cm^{-1} /A1656 cm^{-1}) of WT and mutant myoglobin at different incubation times. Boxplot denote the median (center line), interquartile range (box), whiskers that represents the most extreme data that is no more than 1.5xIQR from the edge of the box and oultiers that are the points outside this range.

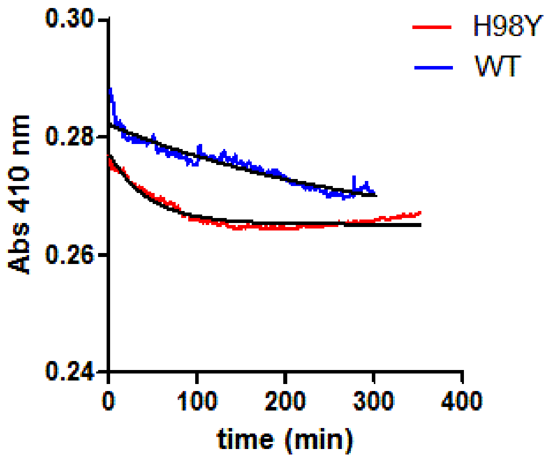

Supplementary Figure 7: Time courses for heme dissociation from WT (blue) and p.His98Tyr (red). Reactions were carried out in 0.2 M phosphate buffer at pH 7.0 with 0.45 M sucrose by mixing WT and p.His98Tyr with p.His65Tyr/Val69Phe apomyoglobin in 1 to10 concentration ratio. Time courses were monitored at 410 nm at 37 °C for 360 min.

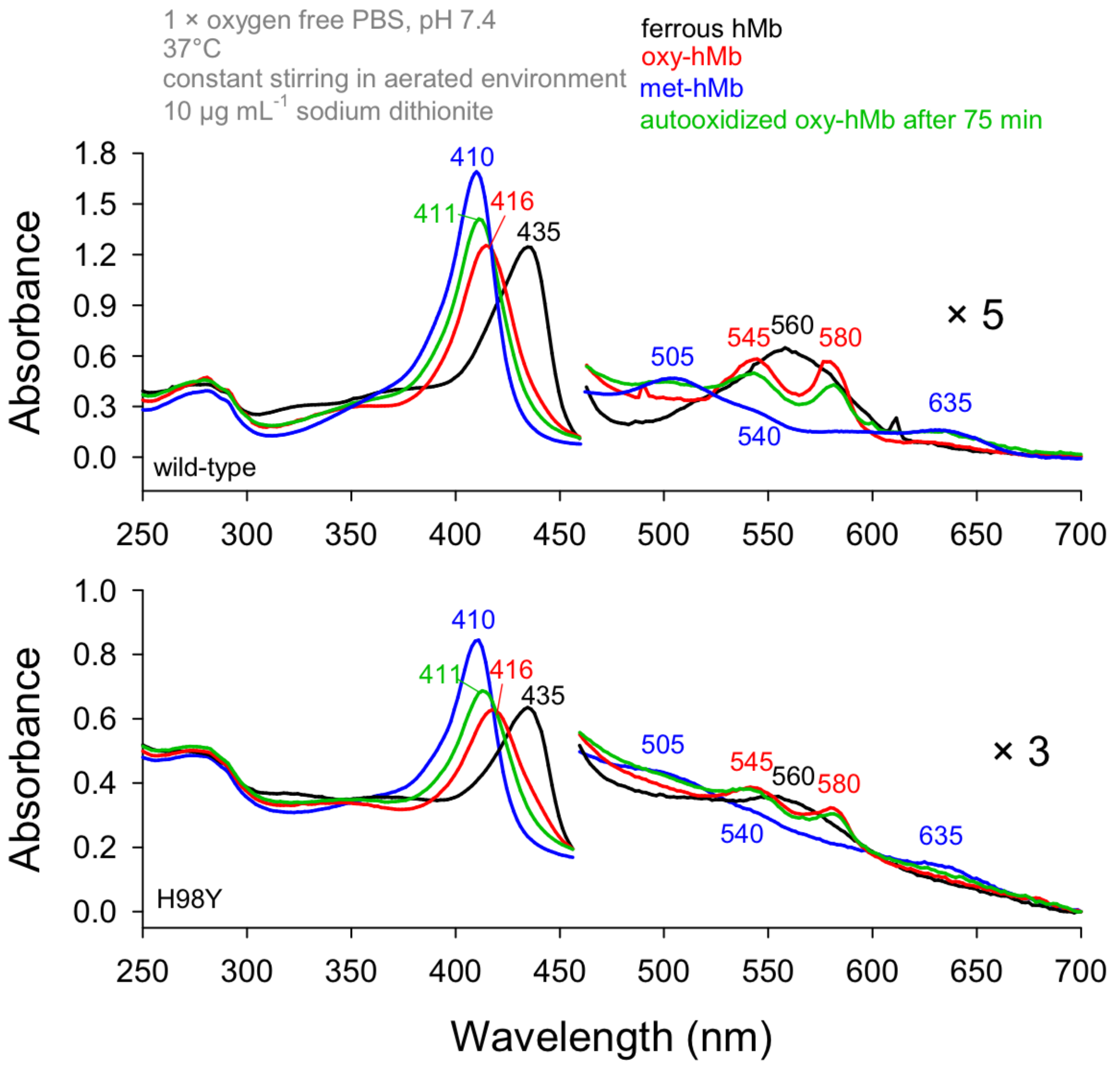

Supplementary Figure 8**:** Spectral features of WT and mutant myoglobin. UV-Vis absorption spectra highlighting changes in spectral features for WT (top) and His98Tyr mutant (bottom) upon transitions from deoxymyoglobin (black) to oxymyoglobin (red) and to metmyoglobin (blue). Spectra were collected in 20 mM phosphate buffer pH 7.4, at 37°C.

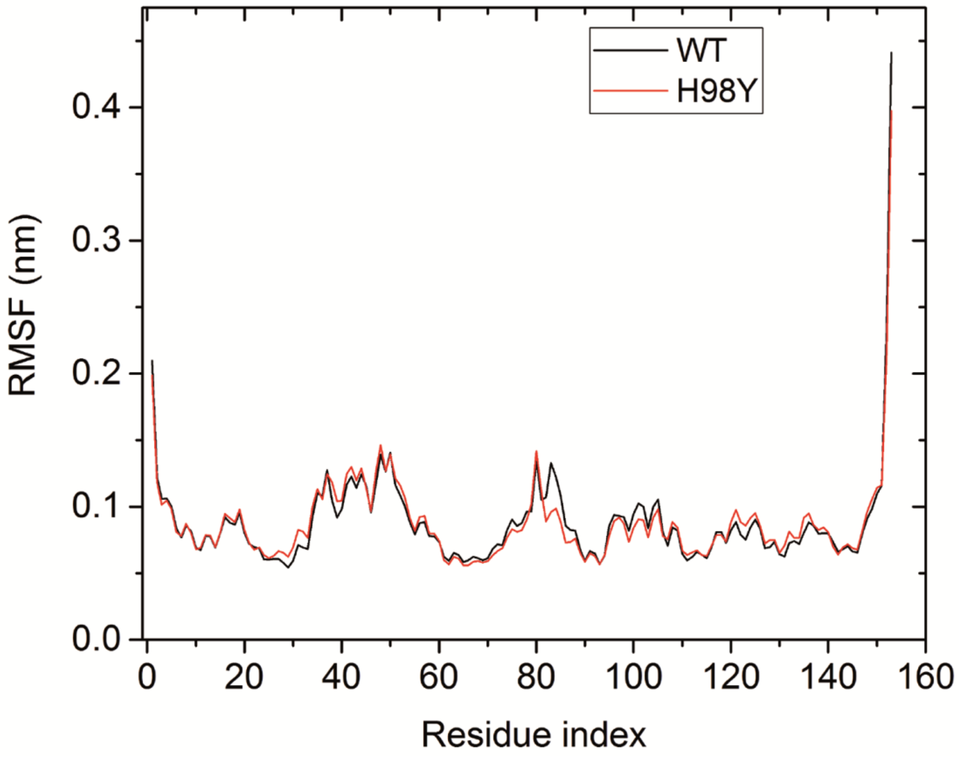

Supplementary Figure 9**:** Protein dynamics of WT and mutant myoglobin. Overlay of the root mean square fluctuations (RMSF) profiles for WT (black) and p.His98Tyr (red) human myoglobin. The RMSF profiles are almost superimposable, indicating that the mutation does not alter significantly the protein dynamics.

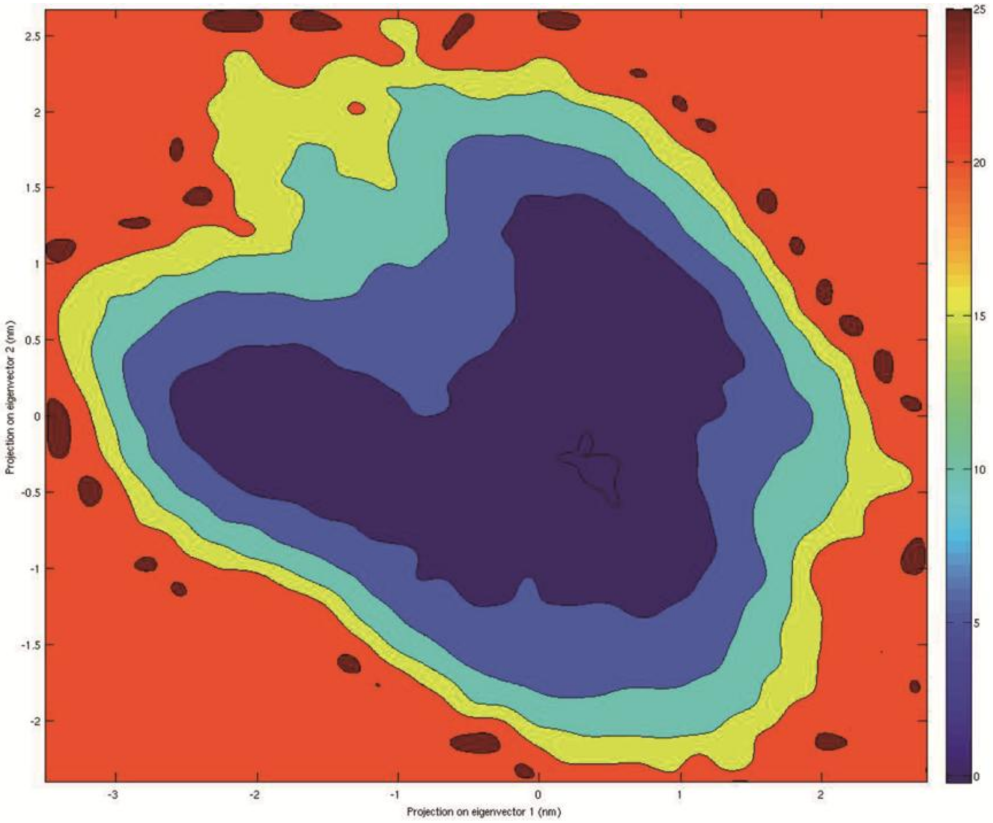

Supplementary Figure 10: Free energy landscapes for WT and mutant myoglobin. Free Energy Landscape (FEL) for the concatenated trajectory obtained by merging the sampled conformations for WT and p.His98Tyr proteins. All the conformations explored by a protein are associated with their corresponding (free) energy level, with the lowest energy levels being the most populated. The energy values associated with each possible distinct conformation are expressed with colors, here ranging from blue (low energy states, therefore high probability) to red (high energy, low probability). The dark blue spot on the plot represents a minimum, associated with low energy, stable conformations. The plot was created by projecting the simulated trajectory obtained by concatenating the individual trajectories obtained by molecular dynamics simulations for both the WT and the p.His98Tyr variant. There is a single minimum, suggesting that the two proteins are characterized by very similar dynamic properties.
